# Systematic Studies on the Effect of Fluorine Atoms in Fluorinated Tolanes on Their Photophysical Properties

**DOI:** 10.3390/molecules26082274

**Published:** 2021-04-14

**Authors:** Masato Morita, Shigeyuki Yamada, Tsutomu Konno

**Affiliations:** Faculty of Molecular Chemistry and Engineering, Kyoto Institute of Technology, Matsugasaki, Sakyo-ku, Kyoto 606-8585, Japan; kit.fusso.201602@gmail.com (M.M.); konno@kit.ac.jp (T.K.)

**Keywords:** aggregation-induced emission enhancement, fluorine, fluorescence, photoluminescence, tolane

## Abstract

In this study, we synthesized a series of fluorinated and non-fluorinated tolanes, in which one or more fluorine atoms were systematically introduced into one aromatic ring of a tolane scaffold, and systematically evaluated their photophysical properties. All the tolanes with or without fluorine substituents were found to have poor photoluminescence (PL) in tetrahydrofuran (THF) solutions. On the other hand, in the crystalline state, non-fluorinated and fluorinated tolanes with one or four fluorine atoms were less emissive, whereas fluorinated tolanes with three or five fluorine atoms exhibited high PL efficiencies (Ф_PL_) up to 0.51. X-ray crystallographic analyses of the emissive fluorinated tolanes revealed that the position of the fluorine substituent played a key role in achieving a high Ф_PL_. Fluorine substituents at the ortho (2/6) and para (4) positions led to tight and rigid packing due to plural π–π stacking and/or hydrogen bonding interactions, resulting in enhanced Ф_PL_ caused by the suppression of non-radiative deactivation. Additionally, fluorinated tolanes with three fluorine atoms exhibited notable aggregation-induced PL emission enhancement in THF/water mixed solvents. This demonstrates that the PL characteristics of small PL materials can be tuned depending on the usage requirements.

## 1. Introduction

Tolanes, which consist of two benzene rings connected with alkyne moiety, have drawn enormous interest as promising small-sized functional molecules owing to their broad applications, such as two-photon absorbers [1,2,3], liquid crystals [4,5,6], light-activated DNA cleavers [7,8], and organic semiconductors [9]. Small-sized solid-state luminescent materials are also needed for practical applications in light-emitting diodes or lighting devices, whereas it is known that tolanes do not emit photoluminescence (PL) because they undergo excitation via the “dark” *trans*-bent πσ* excited states [10,11,12,13,14].

**Figure 1:**
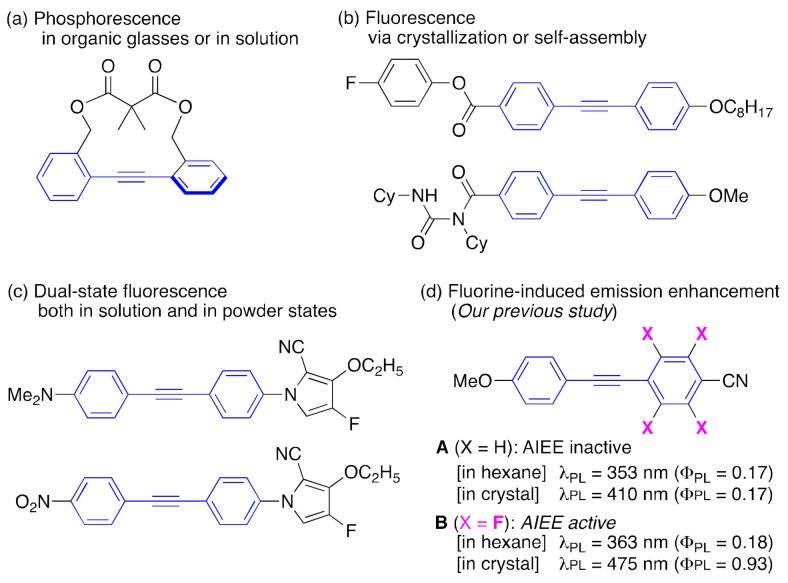
Tolane-based luminescent molecules reported in the literature: (**a**) twist tolanes showing phosphorescence in organic glasses or in solution, (**b**) donor-π-acceptor-type tolanes displaying fluorescence via crystallization or self-assembly, (**c**) tolanes exhibiting dual-state fluorescence, and (**d**) non-fluorinated or fluorinated tolanes-based fluorophores. Abbreviation: AIEE: aggregation-induced emission enhancement.

As a result of extensive photophysical investigations of tolane and the derivatives in recent years, significant advances have been achieved in developing tolane-based compounds that exhibit unique luminescence phenomena. Examples of such innovations include the tethering of two benzene rings of tolane, which induces the emission of phosphorescence in organic glass or solution states (Figure 1a) [15,16], crystallization or the formation of molecular aggregates of tolane derivatives (Figure 1b), which emit fluorescence, and enhancement of the fluorescence of tolanes by suppressing internal conversion from ππ* to the “dark” *trans*-bent πσ excited states [17,18]. Additionally, tolanes bearing fluoropyrrole groups exhibit dual-state fluorescence, emitted both in the solution and powder states (Figure 1c) [19].

Over the last couple of years, our group has explored the synthesis and properties of fluorinated PL molecules because fluorine atoms incorporated into organic structures play a crucial role in the formation of ordered molecular aggregates. The unique characteristics of fluorine atom that contribute to this effect include its (i) large electronegativity, higher than that of all the other elements; and (ii) small atomic size, second only to the size of the hydrogen atom, etc. [20,21]. Our extensive efforts have led to the successful development of fluorinated tolane-based PL molecules [22,23,24]. From our previous results, fluorinated tolanes were found to possess both crystallization-induced emission enhancement (CIEE) as well as aggregation-induced emission enhancement (AIEE) characteristics (Figure 1d) [23,24]. A deep investigation using X-ray crystallographic analyses has revealed that fluorinated tolane **B** in the crystalline state exhibits enhanced PL properties, while the non-fluorinated tolane **A** counterpart does not, owing to the tight and rigid molecular packing through several intermolecular hydrogen bonds, which are needed to suppress the non-radiative deactivation process.

**Figure 2:**
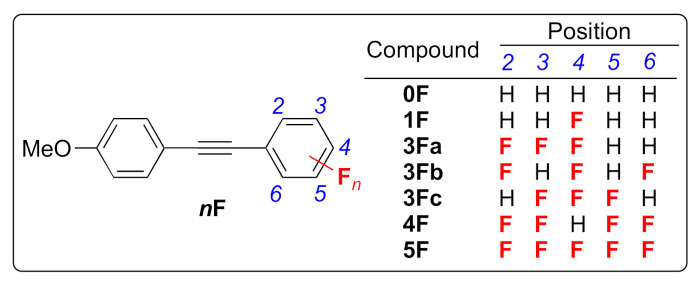
Molecular structures of the fluorinated tolanes used in this study.

To further elucidate the effect of factors such as the number of fluorine atoms and positions of the fluorine substituents on the PL characteristics, we systematically synthesized tolane derivatives **0F**–**4F** with a systematic arrangement of the number and position of fluorine substituents, as shown in Figure 2. In this paper, we demonstrate and discuss the photophysical behavior of **0F**–**4F**. In addition, the photophysical behavior and molecular aggregated structures in the crystalline state of **5F**, which contains five fluorine atoms, are discussed and compared with other such materials in detail, although some photophysical behaviors of **5F** in solution and amorphous states have been reported previously [24].

## 2. Results and Discussion

Based on the synthesis protocol reported previously for **5F** [24], tolane **0F** (without fluorine substituents) and **1F–4F** (with fluorine substituents) were prepared via a Pd(0)-catalyzed Sonogashira cross-coupling reaction using commercially available 4-ethynylanisole and various non-fluorinated or fluorinated aromatic halides. Yields in the range of 46–94% were achieved (Figure S1). In order to assess the photophysical behavior of the compounds in the crystalline state, tolanes **0F**–**5F** were crystallized through double purification by column chromatography, followed by recrystallization. Based on several spectroscopic studies, the tolane **0F**–**5F** crystals were determined to be adequately pure to evaluate photophysical properties such as ultraviolet–visible light (UV–vis) absorption and PL behavior, both in dilute solutions and the crystalline state (Figures S2–S15).

To investigate the effect of fluorine substituents introduced into the tolane scaffold on the photophysical behavior, we initially attempted to measure UV–vis absorption and PL for dilute solution samples, which were prepared by dissolving tolane crystalline powder in tetrahydrofuran (THF) to achieve a 1.0 × 10^−5^ mol L^–1^ concentration. The UV–vis and PL spectra obtained for these samples are shown in Figure 3 and Figures S16–S27, while the photophysical data are summarized in Table 1 and Table S1.

**Figure 3:**
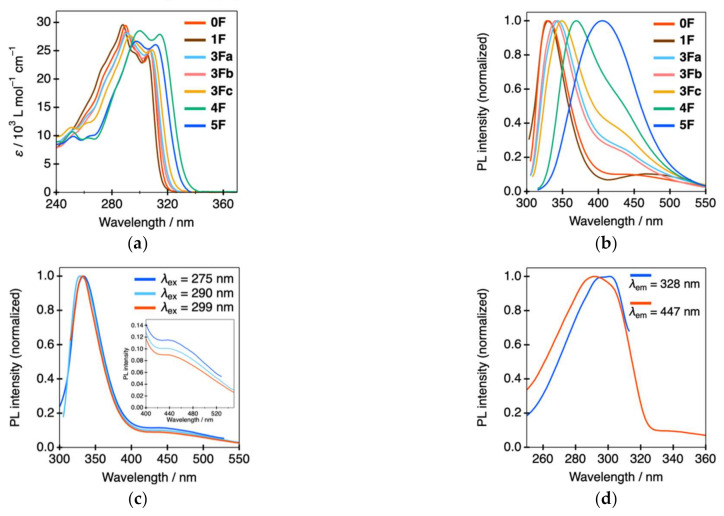
(**a**) UV–vis spectra of **0F**–**5F**; (**b**) photoluminescence (PL) spectra of **0F**–**5F** excited at (λ_abs_) 290 nm for **0F**, 288 nm for **1F**, 291 nm for **3Fa**, 290 nm for **3Fb**, 293 nm for **3Fc**, 300 nm for **4F**, and 297 nm for **5F**; (**c**) PL spectra of **0F** at various excitation wavelengths; and (**d**) excitation spectra of **0F** at 328 and 447 nm.

As shown in Figure 3a, non-fluorinated tolane **0F** and its fluorinated counterparts **1F** and **3Fa**–**c** containing three or fewer fluorine atoms exhibited absorption bands with two maxima (λ_abs_) at approximately 290 and 307 nm. With increasing the number of fluorine substituents, for example, **4F** and **5F** substituted with four and five fluorine atoms, respectively, in one of the aromatic rings of the tolane scaffold, a slight long-wavelength shift in λ_abs_ of approximately 10 nm was observed. Furthermore, the energy gap (Δ*E*) between the highest occupied molecular orbital (HOMO) and lowest unoccupied MO (LUMO), which was experimentally obtained via cyclic voltammetry (CV) measurements (Figure S35 and Table S3), decreased with the increasing number of fluorine substituents. Therefore, the red-shift in λ_abs_ may be attributable to the decreased HOMO–LUMO energy gap, Δ*E*. Additionally, the calculated data involving dipole moment in long molecular axis, HOMO and LUMO energies obtained from DFT calculations are also summarized in Table S6.

As shown in Figure 3b, in all the compounds, the maximum PL wavelength (λ_PL_) was 328–406 nm. In addition to the intense PL band observed in the short-wavelength region, interestingly, **0F** and **1F** exhibited a weak PL band around 450 nm. The two diphenylacetylene emission bands have been reported to originate from radiative deactivation via a ππ* state for the short-wavelength band and a dark πσ* state for the long-wavelength band [14,25]. Furthermore, **3F** and **4F** exhibited a major PL band with **λ**_PL_ between 340 and 370 nm, accompanied by a shoulder peak with **λ**_PL_ at approximately 430 nm. In contrast, **5F** containing five fluorine substituents was found to exhibit a single PL band with λ_PL_ at 406 nm. To gain more insights into these two PL bands, the excitation wavelength (λ_ex_)-dependent PL behavior was investigated using **0F** as an example (Figure 3c). Excitation by higher energy light caused a gradual increase in the PL intensity in the long-wavelength region compared with the PL intensity observed upon excitation by lower energy light. In addition, as shown in Figure 3d, the excitation spectra observed at **λ**_PL_ at 447 nm were slightly blue-shifted in comparison with the excitation spectra observed at 328 nm. This PL behavior likely originates from increased internal conversion from a higher-order excited state to a dark *trans*-bent excited state [14]. As a result of this major internal conversion process, the PL efficiency (Ф_PL_) for **0F**–**3F** appears to be extremely low (<0.01). In the case of **4F** and **5F**, a slight increase in Ф_PL_ (0.04 and 0.08 for **4F** and **5F**, respectively) was observed, owing to major contributions of the fast radiative process from the charge-transfer state, with the exception of a minor non-radiative process from the dark *trans*-bent excited state. In order to assess the radiative process for the tolane derivatives, we tested their PL lifetimes (τ_PL_). The τ values are depicted in Figure S33 and the data are also listed in Table 1 and Table S2. The τ_PL_ values of the THF solution containing **0F** at λ_PL_ of 328 nm were approximately 0.82 and 5.08 ns. The observed τ_PL_ values were found to be different from those reported [25,26], which is likely due to the change of solvent polarity [27,28]. Compound **0F** in THF solution exhibited fluorescence due to light emission from two components at the singlet S_1_ excited state. Similarly, the **1F**–**5F** compounds also exhibited fluorescence, which stemmed from two luminescent components at the S_1_ excited states.

**Figure 4:**
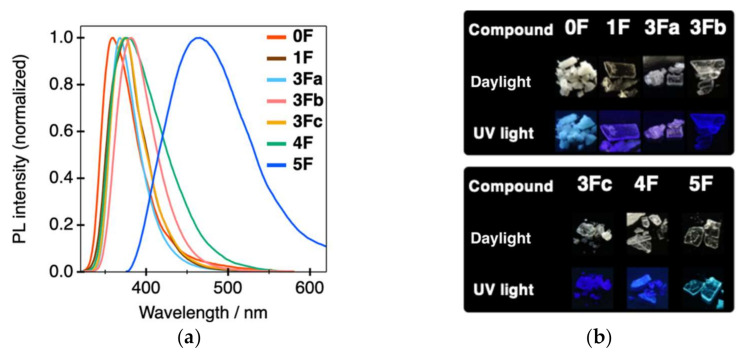
(**a**) PL spectra of **0F**–**5F** in the crystalline states (λ_ex_ = 300 nm for **0F**–**4F** and 360 nm for **5F**). (**b**) Photographs under daylight and UV light (λ_ex_ = 365 nm).

We previously reported that tolane **5F** containing a pentafluorobenzene scaffold exhibited weak PL (Ф_PL_ = 0.14) in the amorphous state [24]. After extensive trials, we were ultimately successful in producing single crystals of **5F**. To our delight, we found that the PL efficiency of crystalline **5F** was four-fold higher (Ф_PL_ = 0.51) compared to that of amorphous **5F**. Based on these results, we focused on the PL characteristics of a series of tolane compounds **0F**–**5F** in the crystalline-state. Figure 4 and Figure S32 shows the PL spectra and photographs obtained under both daylight and UV light conditions (λ_ex_ = 365 nm). The photophysical data obtained are summarized in Table 2.

Crystalline **5F** exhibited a single band showing light-blue PL at λ_PL_ of around 465 nm, whereas crystalline samples of **0F**–**4F**, which were prepared by recrystallization from CH_2_Cl_2_/MeOH (*v*/*v* = 1/1), were found to exhibit deep-blue PL with λ_PL_ in the 359–381 nm range. As mentioned above, the Ф_PL_ of **5F** was as high as 0.51, whereas the corresponding values for tolanes **0F**, **1F**, and **4F** were observed to be quite low (0.04, 0.10, and 0.04 for **0F**, **1F**, and **4F**, respectively). Considering the Ф_PL_ of a series of **3F** containing three fluorine atoms at different substitution positions on the benzene ring, it is interesting that the Ф_PL_ was significantly affected by the position of the fluorine substituents. For example, **3Fa** with three fluorine atoms at the 2, 3, and 4 positions and **3Fb** with three fluorine atoms at the 2, 4, and 6 positions exhibited relatively high Ф_PL_ (up to 0.37), whereas **3Fc** with three fluorine atoms at the 3, 4, and 5 positions exhibited low Ф_PL_ (0.14).

**Figure 5:**
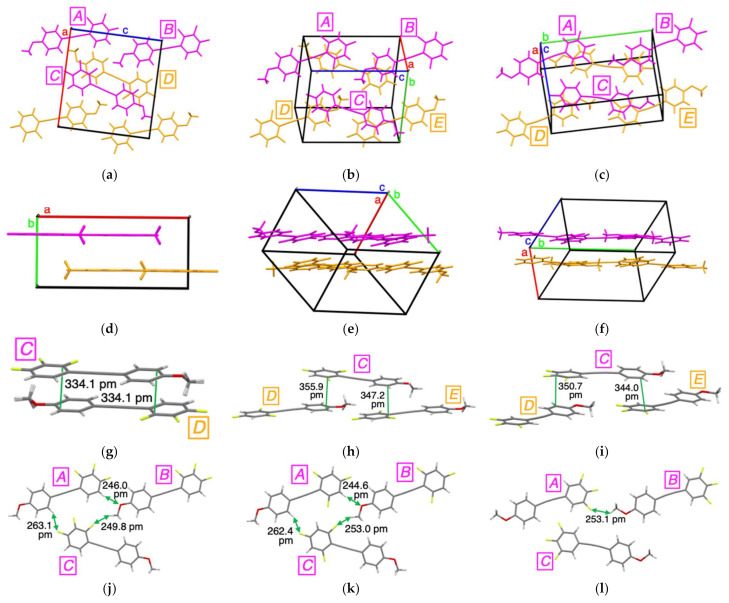
Crystal structure of (**a**,**d**) **3Fa**, (**b**,**e**) **3Fb,** and (**c**,**f**) **3Fc**. Intermolecular interactions in (**g**,**j**) **3Fa**, (**h**,**k**) **3Fb,** and (**i**,**l**) **3Fc**.

To understand why the Ф_PL_ values for **3Fa**–**c** and **5F** were higher than those for **0F**, **1F**, and **4F**, we performed X-ray crystallographic analyses for crystalline **3Fa**–**c**, **4F**, and **5F**, which were successfully obtained by recrystallization from a mixed solvent system containing CH_2_Cl_2_/MeOH. Figure 5 shows the crystal packing structures of **3Fa**–**c** and the crystallographic data are summarized in Table S4. 

Tolanes **3Fa**–**c** containing three fluorine substituents were found to possess similar packing structures with four molecular units in a unit cell, in which three molecules, ***A*** to ***C***, existed in-plane (Figure 5a–f). Considering the intermolecular π–π stacking interactions of **3Fa**–**c**, **3Fa** exhibited two π–π stacking interactions between molecules π***_C_***···π***_D_*** with 334.1 pm of interlayer distance on a one-to-one basis (Figure 5g). As shown in Figure 5h,i, in contrast, **3Fb** and **3Fc** possessed two π–π stacking interactions between two molecules π***_C_***···π***_D_***/π***_C_***···π***_E_*** with interlayer distances of 355.9 and 347.2 pm for **3Fb** and 350.7 and 344.0 pm for **3Fc**, respectively [29,30]. In addition, in **3Fa** and **3Fb,** three hydrogen bonding interactions were observed among the three molecules in the plane: H***_A_***···O***_B_***/H***_B_***···F***_C_***/F***_C_***···H***_A_*** (Figure 5j,k). In contrast, **3Fc** possessed only one F***_A_***···H***_B_*** hydrogen bond without any other hydrogen bonding interactions (Figure 5l). Furthermore, the nonradiative rate constant (*k*_nr_) values, which were calculated from τ_PL_ in crystal (Figure S34), for **3Fa** and **3Fb** were approximately one-half or one-fifth of the corresponding value for **3Fc**. These results clearly indicate that the intermolecular π–π stacking and hydrogen bonding interactions resulted in the formation of tight and rigid packing structures in the crystalline state, which likely suppresses nonradiative deactivation through molecular motions and results in strong PL in the crystal. Furthermore, **3Fa** and **3Fb** with three hydrogen bonding interactions exhibited higher Ф_PL_ compared to **3Fc,** which had one hydrogen bond.

**Figure 6:**
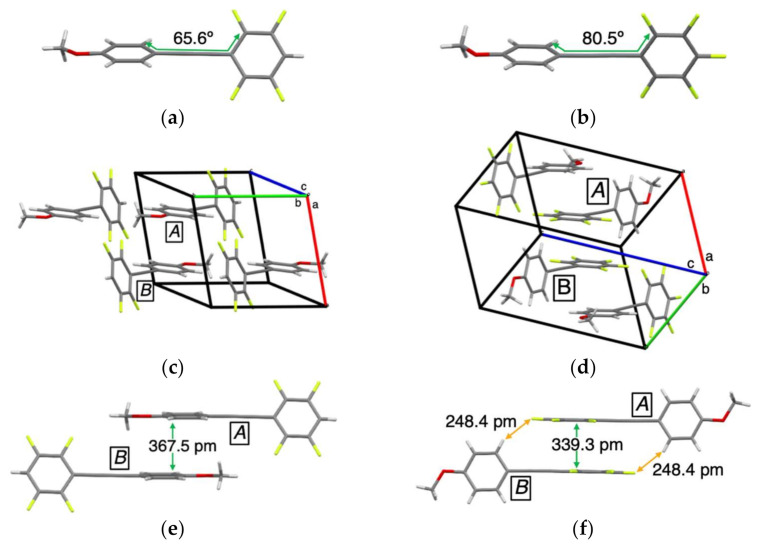
Crystal structures of (**a**) **4F**, (**b**) **5F**, packing structures of (**c**) **4F**, (**d**) **5F**, and intermolecular interactions of (**e**) **4F**, (**f**) **5F**.

Figure 6 also shows the results of X-ray crystallographic analyses for **4F** and **5F**, in which the crystallographic data are summarized in Table S5. Tolane **4F** containing four fluorine atoms at the 2, 3, 5, and 6 positions was found to have a twisted structure with a dihedral angle of 65.6° between the two aromatic rings connected to the alkyne moiety (Figure 6a). In the packing structures, two molecules were present in the unit cell (Figure 6c). One π–π intermolecular interaction was observed between the ***A***···***B*** molecular units, with the closest interatomic distance (π***_A_***···π***_B_***) being 367.5 pm (Figure 6e).

Similarly, the crystal structure of **5F** containing five fluorine atoms was also found to be twisted with a dihedral angle of 80.5° between the two aromatic rings connected with the alkyne moiety; four molecular units were present in a unit cell (Figure 6b,d). Two molecular units ***A***···***B*** that existed in the central position were tightly held in place via a π–π stacking interaction (π***_A_***···π***_B_*** = 339.3 pm) and two hydrogen bonding interactions (F_Ar_···H_Ar_ = 248.4 pm). The H···F distances in the hydrogen bonds were observed to be much shorter than those in **3Fa**–**c**, resulting in tighter and more rigid structures and the independent formation of dimer units. Owing to the tight dimer formation through multiple intermolecular interactions, the *k*_nr_ of **5F** (2.07 × 10^8^ s^−1^) was found to be one-sixth of the *k*_nr_ of **0F** or **4F**. It can be concluded that the PL emission of **5F** in the crystal is likely to shift to the long-wavelength region and exhibit higher PL efficiency, compared to the other analogs. Judging from the relationship between the crystal structure and PL efficiency, the incorporation of fluorine substituents at either 2/6 (*ortho*) and/or 4 (*para*) positions appears to be essential for efficient PL emission in the crystalline states.

**Figure 7:**
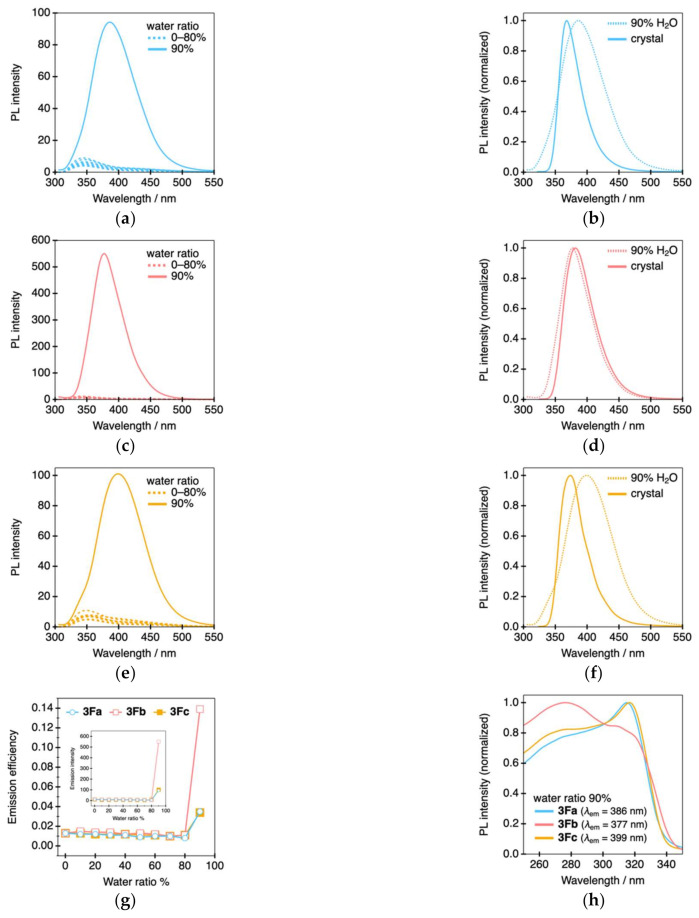
PL spectra of (**a**) **3Fa**, (**c**) **3Fb**, and (**e**) **3Fc** in THF/water mixed solvent (λ_ex_ = 290 nm). Differences in the PL spectra between the molecular aggregates obtained in the THF/water system and crystalline states for (**b**) **3Fa**, (**d**) **3Fb**, and (**f**) **3Fc**. (**g**) Relationship between PL efficiency and additional water ratio (inset: relationship between PL intensity and additional water ratio). (**h**) Excitation spectra of the molecular aggregates for **3Fa**–**c** obtained after the addition of 90% water.

Considering the Ф_PL_ change for **3Fa**–**c** in the solution and crystalline states, we tested their AIEE characteristics [31,32,33]. The PL spectra are shown in Figure 7 and Figures S28–S32.

As shown in Figure 7a,c,e, the PL intensities of **3Fa**–**c** did not change at all with up to 80% water addition, whereas the PL intensities significantly increased upon the addition of 90% water. In **3Fb**, Ф_PL_ markedly improved to 0.14 when the water amount reached 90%, whereas Ф_PL_ only slightly improved to 0.04 and 0.03 in **3Fa** and **3Fc** (Figure 7g), respectively, which suggests that the molecular aggregates formed in the solution upon water addition depend on the substitution position of the fluorine atoms. Furthermore, as shown in Figure 7h, this is also supported by the fact that only **3Fb** exhibited a different excitation spectrum compared to **3Fa** and **3Fc** at 90% water content. Comparing the PL spectral shapes for the molecular aggregates formed in THF/water mixtures with those formed in the crystalline state, the λ_PL_ values for **3Fa** and **3Fc** were slightly red-shifted in the THF/water mixtures, whereas in the crystalline state, only **3Fb** exhibited a similar spectral shape (Figure 7b,d,f). The change in the spectral shape likely originates from the changes in the molecular aggregated structures, which clearly indicate that the PL characteristics can be modulated by altering the molecular aggregates.

## 3. Materials and Methods

### 3.1. Materials

The ^1^H-NMR (400 MHz) and ^13^C-NMR (100 MHz) spectra were obtained using an AVANCE III 400 NMR spectrometer (Bruker, Rheinstetten, Germany) in chloroform-*d* (CDCl_3_) solution, and the chemical shifts are reported in parts per million (ppm) using the residual protons in the NMR solvent. The ^19^F-NMR (376 MHz) spectra were obtained using an AVANCE III 400 NMR spectrometer (Bruker, Rheinstetten, Germany) in CDCl_3_ solution with CFCl_3_ (δ_F_ = 0 ppm) as an internal standard. Infrared (IR) spectra were recorded using the KBr method with an FTIR-4100 type A spectrometer (JASCO, Tokyo, Japan). All the spectra are reported in terms of wavenumber (cm^–1^). High-resolution mass spectra (HRMS) were recorded on a JMS700MS spectrometer (JEOL, Tokyo, Japan) using the fast atom bombardment (FAB) method. All the chemicals, including solvents, were of reagent grade and were purified in the usual manner prior to use. Column chromatography was carried out on silica gel (FUJIFILM Wako Pure Chemical Corporation, Wakogel^®^ 60 N, 38–100 μm) and thin-layer chromatography (TLC) was performed on silica gel TLC plates (Merck, Silica gel 60F_254_; Kenilworth, NJ, USA).

### 3.2. General Synthesis Procedure for the Pd(0)-Catalyzed Sonogashira Cross-Coupling Reaction

In a flask, an aromatic halide, 4-ethynylanisole, dichlorobis(triphenylphosphine)palladium(II), triphenylphosphine, copper(I) iodide, and triethylamine, and the suspended solution were stirred at 60 °C overnight. After the reaction times indicated, the precipitate formed during the reaction was separated by atmospheric filtration, while the filtrate was poured into a saturated aqueous ammonium chloride solution. The crude product was extracted with ethyl acetate (EtOAc) three times, and the combined organic layer was washed once with brine. The collected organic layer was dried over anhydrous Na_2_SO_4_, which was separated by filtration. The filtrate was evaporated in vacuo and subjected to silica gel column chromatography (eluent: hexane/EtOAc = 20/1), followed by recrystallization from CH_2_Cl_2_/MeOH (*v*/*v* = 1/1), to obtain the desired product in a 46–94% yield.

#### 3.2.1. [2-(4-methoxyphenyl)ethyn-1-yl]benzene (**0F**)

Yield: 94% (White solid); m.p.: 59.1–60.3 °C; ^1^H-NMR (CDCl_3_): δ 3.83 (s, 3H), 6.88 (d, *J* = 8.9 Hz, 2H), 7.30–7.36 (m, 3H), 7.46–7.52 (m, 4H). The spectral data were fully in agreement with the reported data [34].

#### 3.2.2. 1-Fluoro-4-[2-(4-methoxyphenyl)ethyn-1-yl]benzene (**1F**)

Yield: 88% (White solid); m.p.: 89.2–91.3 °C; ^1^H-NMR (CDCl_3_): δ 3.84 (s, 3H), 6.88 (d, *J* = 8.9 Hz, 2H), 7.03 (t, *J* = 8.8 Hz, 2H), 7.44–7.50 (m, 4H). The spectral data were fully in agreement with the reported data [35].

#### 3.2.3. 1,2,3-Trifluoro-4-[2-(4-methoxyphenyl)ethyn-1-yl]benzene (**3Fa**)

Yield: 65% (White solid); m.p.: 75.0–76.3 °C; ^1^H-NMR (CDCl_3_): δ 3.84 (s, 3H), 6.89 (d, *J* = 8.9 Hz, 2H), 7.18–7.25 (m, 1H), 7.18–7.25 (m, 1H), 7.49 (d, *J* = 8.9 Hz, 2H); ^19^F-NMR (CDCl_3_): δ–131.16 (ddd, *J* = 20.3, 8.6, 6.8 Hz, 1F), –133.15 to –133.04 (m, 1F), –160.17 (tdd, *J* = 20.4, 6.7, 2.1 Hz, 1F). The spectral data were fully in agreement with the reported data [36].

#### 3.2.4. 1,3,5-Trifluoro-4-[2-(4-methoxyphenyl)ethyn-1-yl]benzene (**3Fb**)

Yield: 46% (White solid); m.p.: 96.5–97.5 °C; ^1^H-NMR (CDCl_3_): δ 3.84 (s, 3H), 6.68–6.75 (m, 2H), 6.89 (d, *J* = 8.9 Hz, 2H), 7.51 (d, *J* = 8.9 Hz, 2H); ^13^C-NMR (CDCl_3_): δ 55.4, 73.9, 99.1–99.2 (m), 101.9 (dd, *J* = 19.8, 4.9 Hz), 100.3–100.8 (m), 114.2, 114.6, 133.4, 160.3, 162.1 (dt, *J* = 250.5, 14.6 Hz), 163.3 (dq, *J* = 252.6, 7.7 Hz); ^19^F-NMR (CDCl_3_): δ –105.24 (t, *J* = 7.5 Hz, 2F), –106.49 to –106.41 (m, 1F); IR (KBr): ν 3095, 3077, 2841, 2362, 2223, 1884, 1636, 1590, 1520, 1440, 1250, 1031, 827 cm^–1^; HRMS: (FAB+) *m*/*z* [M]^+^ calcd for C_15_H_9_F_3_O: 262.0605; found: 262.0604.

#### 3.2.5. 1,2,6-Trifluoro-4-[2-(4-methoxyphenyl)ethyn-1-yl]benzene (**3Fc**)

Yield: 59% (White solid); m.p.: 66.8–67.9 °C; ^1^H-NMR (CDCl_3_): δ 3.84 (s, 3H), 6.88 (d, *J* = 8.9 Hz, 2H), 7.11 (dd, *J* = 8.2, 6.6 Hz, 2H), 7.45 (d, *J* = 8.9, 2H); ^13^C-NMR (CDCl_3_): δ 55.4, 85.3 (dd, *J* = 6.0, 3.0 Hz), 91.2 (d, *J* = 2.0 Hz), 114.2, 114.3, 115.8 (dd, *J* = 16.2, 6.3 Hz), 119.8 (ddd, *J* = 15.2, 10.2, 5.2), 133.3, 140.1 (dt, *J* = 252.8, 15.3), 151.11 (ddd, *J* = 248.7, 10.2, 4.5 Hz), 160.3; ^19^F-NMR (CDCl_3_): δ –134.86 (dd, *J* = 20.4, 8.0 Hz, 2F), –160.04 (tt, *J* = 20.5, 6.4 Hz, 1F); IR (KBr): ν 3098, 3023, 2845, 2360, 2343, 2213, 1608, 1527, 1508, 1430, 1252, 1044, 832 cm^–1^; HRMS: (FAB+) *m*/*z* [M]^+^ calcd for C_15_H_9_F_3_O: 262.0605; found: 262.0604.

#### 3.2.6. 2,3,5,6-Tetrafluoro-4-[2-(4-methoxyphenyl)ethyn-1-yl]benzene (**4F**)

Yield: 65% (White solid); m.p.: 76.8–77.4 °C; ^1^H-NMR (CDCl_3_): δ 3.85 (s, 3H), 6.91 (d, *J* = 8.9 Hz, 2H), 7.02 (ddt, *J* = 17.0, 9.7, 7.3 Hz, 1H), 7.53 (d, *J* = 8.9 Hz, 2H); ^13^C-NMR (CDCl_3_): δ 55.5, 73.5 (dd, *J* = 4.0, 4.0 Hz), 102.3 (dd, *J* = 3.6, 3.6 Hz), 105.5–106.1 (m), 105.7 (dd, *J* = 22.9, 22.9 Hz), 113.8, 114.3, 133.7, 145.9 (dm, *J* = 246.5), 146.7 (dddd, *J* = 250.1, 14.4, 3.5, 3.5 Hz), 160.8; ^19^F-NMR (CDCl_3_): δ –137.63 (dq, *J* = 20.0, 7.4 Hz, 2F), –139.75 (qui, *J* = 11.8 Hz, 2F); IR (KBr): ν 3103, 3079, 2964, 2839, 2361, 2224, 1604, 1491, 1398, 1255, 1173, 1032, 930 cm^–1^; HRMS: (FAB+) *m*/*z* [M]^+^ calcd for C_15_H_8_F_4_O: 280.0511; found: 280.0503.

### 3.3. Photophysical Measurements

UV/vis absorption spectra were recorded on a V-530 absorption spectrometer (JASCO, Tokyo, Japan). PL spectra in the solution and crystal forms were acquired using an FP-6600 fluorescence spectrometer (JASCO, Tokyo, Japan). The absolute quantum yields in solution and crystal forms were measured using the Quantaurus-QY measurement system C11347-01 (Hamamatsu Photonics, Hamamatsu, Japan). The PL lifetime was obtained using a Quantaurus-Tau fluorescence lifetime spectrometer C11367-34 (Hamamatsu Photonics, Hamamatsu, Japan).

### 3.4. Single Crystal X-ray Diffraction

Single crystal X-ray diffraction spectra were recorded on an XtaLAB AFC11 diffractometer (Rigaku, Tokyo, Japan). The reflection data were integrated, scaled, and averaged using CrysAlisPro program (ver. 1.171.39.43a, Rigaku Corporation, Akishima, Japan) [37]. Empirical absorption corrections were applied using the SCALE 3 ABSPACK scaling algorithm (CrysAlisPro). The structures were identified by a direct method (SHELXT-2018/2 [38]) and refined using a full matrix least squares method (SHELXL-2018/3 [39]) visualized by Olex2 [40]. The crystallographic data were deposited into the Cambridge Crystallographic Data Centre (CCDC) database (CCDC 2070935 for **3Fa**, 2070936 for **3Fb**, 2070937 for **3Fc**, 2070938 for **4F** and 2070939 for **5F**). These data can be obtained free of charge from the CCDC via www.ccdc.cam.ac.uk/data_request/cif (accessed on 13 April 2021).

### 3.5. Cyclic Voltammetry

Cyclic voltammetry (CV) measurements were carried out using an ECstat-101 potentiostat (EC frontier, Kyoto, Japan) with glassy carbon, Pt, and Ag/AgCl as the working, counter, and reference electrodes, respectively. Ferrocene (Fc)/ferrocenium (Fc^+^) was used as an external reference, while tetrabutylammonium hexafluorophosphate (Bu_4_NPF_6_) was used as the supporting electrolyte (0.1 mol L^−1^). All the measurements were performed after argon bubbling for 30 min in 1 × 10^−3^ mol L^−1^ acetonitrile solution, with a scan rate of 50 mV s^−1^. HOMO and LUMO energy levels were estimated from the onset potentials of the oxidation (*E_Ox_*) and reduction (*E_Red_*) waves (versus Fc/Fc^+^) using the following equation: *E*_HOMO_ = −4.80 − *E*_Ox_, *E*_LUMO_ = −4.80 − *E*_Red_, *ΔE* = *E*_LUMO_ − *E*_HOMO_.

## 4. Conclusions

To gain insights into the structure–property relationships of fluorinated tolanes, we synthesized various tolanes with and without fluorine substituents (**0F**–**5F**). We evaluated the photophysical properties and crystal structures of these compounds in detail. During extensive investigations, all the derivatives were found to be non-emissive in dilute THF solution, with tight molecular packing structures formed via π–π stacking as well as hydrogen bonding interactions. Interestingly, tight molecular aggregates were formed when the fluorine substituents were incorporated at the ortho and para positions. The fluorinated tolanes containing fluorine substituents at these positions were found to emit PL efficiently, resulting in Ф_PL_ values in the 0.31–0.51 range. The range of PL behavior exhibited in solution (non-emissive) and in the crystalline state (emissive) piqued our interest to study the aggregation-induced emission enhancement characteristics. From the PL investigations of **3F** (containing three fluorine substituents) in THF/water mixtures, we found a significant enhancement in the PL intensity upon adding 90% water, although the PL intensity was low, at approximately 0.01, when the amount of water was below 80%. The PL spectral shape was different from that in the crystalline state, obtained by recrystallization. It was found that the PL characteristics of fluorinated tolane **3F** could be tuned by altering the molecular aggregates, which is promising for fabricating materials with tunable PL properties.

## Figures and Tables

**Table 1:** Photophysical properties of tolanes **0F**–**5F** in dilute THF solution (concentration: 1.0 × 10^–5^ mol L^–1^).

Compound	E_HOMO_ (eV) ^a^	E_LUMO_ (eV) ^a^	Δ*E* (eV) ^a^	λ_abs_ (nm) [*ε*, 10^3^ L·mol^–1^·cm^–1^]	λ_PL_ (nm) ^b^	Ф_PL_^c^	*τ*_ave_ (ns) ^d^	*τ*_1_ (ns) ^d^	*τ*_2_ (ns) ^d^
**0F**	−5.81	–2.09	3.72	290 [29.5], 299 [24.6], 307 [25.0]	328, 447	<0.01	2.63	0.82	5.08
**1F**	–5.81	–2.06	3.75	288 [29.6], 297 [24.5], 305 [24.2]	330, 469	<0.01	2.86	0.78	5.23
**3Fa**	–5.96	–2.33	3.63	291 [28.2], 307 [25.0]	343, 431	0.01	2.55	0.86	4.84
**3Fb**	–5.96	–2.27	3.69	290 [28.5], 307 [25.3]	340, 433	0.01	2.12	0.75	4.92
**3Fc**	–5.96	–2.38	3.58	293 [27.7], 309 [25.2]	349, 436	0.01	2.37	0.86	5.93
**4F**	–6.07	–2.50	3.57	300 [28.5], 314 [27.9]	369, 432	0.04	3.38	1.12	5.68
**5F**	–6.02	–2.61	3.41	297 [26.7], 311 [26.1]	406	0.08	2.82	1.41	5.42

^a^ Determined by cyclic voltammetry measured in 1.0 × 10^–3^ mol L^–1^ acetonitrile solution. ^b^ Excitation by UV light at λ_abs_. ^c^ An integrating sphere was used. ^d^ PL lifetime (τ) monitored PL light at λ_PL_. τ_ave_: average of PL lifetime, τ_1_: PL lifetime for the first excited component and τ_2_: for the second excited component.

**Table 2:** Photophysical properties of tolanes **0F**–**5F** in the crystalline states.

Compound	λ_PL_ (nm) ^a^	Ф_PL_^b^	τ_PL_ (ns) ^c^	*k*_r_ (10^8^ s^−1^) ^d^	*k_n_*_r_ (10^8^ s^−1^) ^e^
**0F**	359	0.04	0.76	0.53	12.6
**1F**	375	0.10	2.21	0.45	4.07
**3Fa**	368	0.31	2.12	1.46	3.25
**3Fb**	381	0.37	3.81	0.97	1.65
**3Fc**	374	0.14	1.13	1.24	7.61
**4F**	375	0.04	0.77	0.52	12.5
**5F**	465	0.51	2.37	2.15	2.07

^a^ λ_ex_ = 300 nm for **0F**–**4F** and 360 nm for **5F**. ^b^ An integrating sphere was used. ^c^ Monitored PL at λ_PL_. ^d^ Radiative rate constant: *k*_r_ = Ф_PL_/τ_PL_. ^e^ Non-radiative rate constant: *k*_nr_ = (1 − Ф_PL_)/τ_PL_.

## Data Availability

Data is contained within the article or supplementary material.

## Supplementary Materials

The following are available online, Figure S1: Synthetic method of the target compounds; Figures S2–S15: NMR spectra of new compounds; Figures S16–S22: UV–vis and PL spectra in THF solution; Figure S23: UV–vis and PL spectra in hexane solution; Figure S24: UV–vis and PL spectra in CH_2_Cl_2_ solution; Figures S25–S27: solvatochromic PL properties of **3Fa**–**c**; Figures S28–S30: PL spectra of **3Fa**–**3Fc** in THF/water mixed solution; Figure S31: photographs of the PL behavior for **3Fa**–**3Fc** in the mixed solution; Figure S32: PL spectra of **0F**–**5F** in crystal; Figures S33 and S34: PL lifetime decay curve; Figure S35: CV curve of **0F**–**5F**; Table S1: photophysical data of **0F**–**5F**; Table S2: PL lifetime of **0F**–**5F** in THF solution; Table S3: Electrical properties obtained by CV measurement; Tables S4 and S5: crystallographic data of **3Fa**–**3Fc**, **4F** and **5F**; Table S6: Results of DFT calculations.

## Sample Availability

Samples of all compounds are available from the authors.
