# Supplementary material for: Systematic Studies on the Effect of Fluorine Atoms in Fluorinated Tolanes on Their Photophysical Properties
Source: Molecules. 2021 Apr 14;26(8):2274. doi: 10.3390/molecules26082274 (PMC8070916; doi:10.3390/molecules26082274)
Supplement: Supplementary file 1 [file molecules-26-02274-s001.pdf]

molecules

MDPI

Supplementary Material

Systematic Studies on the Effect of Fluorine Atoms in Fluorinated Tolanes on Their Photophysical Properties

Masato Morita, Shigeyuki Yamada * and Tsutomu Konno

Faculty of Molecular Chemistry and Engineering, Kyoto Institute of Technology, Matsugasaki, Sakyo-ku, Kyoto 606-8585, Japan; kit.fusso.201602@gmail.com (M.M.), konno@kit.ac.jp (T.K.)
* Correspondence: syamada@kit.ac.jp; Tel.: +80-75-724-7517

Contents

1. Synthesis of **0F–4F**------S-2
2. ^{1}H, ^{19}F, and ^{13}C-NMR spectra of **0F–4F** ------S-3
3. Absorption and PL spectra of THF solution ------S-10
4. Absorption and PL spectra in hexane and dichloromethane------S-12
5. Solvatochromic properties of **3Fa–c**------S-13
6. PL spectra of THF/water mixed solution ------S-14
7. PL spectra of crystal ------S-15
8. PL lifetime of solution and crystal------S-16
9. Cyclic voltammetry (CV) ------S-18
10. Crystallographic data ------S-19
11. DFT calculation------S-20

1. Synthesis of 0F–4F

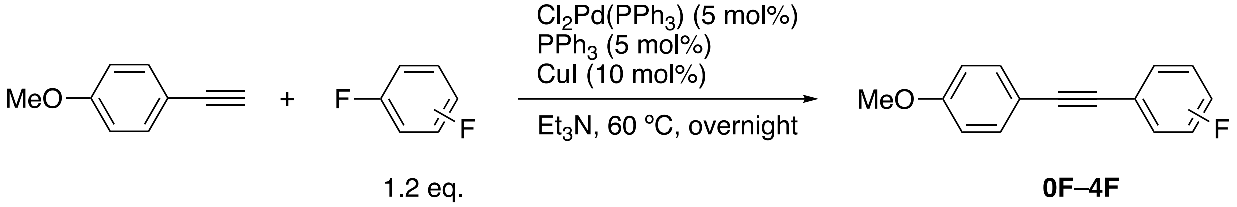

0F–4F

Figure S1. Synthesis of target compound.

In a flask, an aromatic halide, 4-ethynylanisole, dichlorobis(triphenylphosphine)palladium(II), triphenylphosphine, copper(I) iodide, and triethylamine, and the suspended solution were stirred at 60 °C overnight. After the reaction time indicated, the precipitate formed during the reaction was separated by atmospheric filtration, while the filtrate was poured into a saturated aqueous ammonium chloride solution. The crude product was extracted with ethyl acetate (EtOAc) three times and the combined organic layer was washed once with brine. The organic layer collected was dried over anhydrous Na_2SO_4, which was separated by filtration. The filtrate was evaporated in-vacuo and subjected to silica-gel column chromatography (eluent: hexane/EtOAc = 20/1), followed by recrystallization from CH_2Cl_2/MeOH (*v*/*v* = 1/1), to obtain the desired product in 46%–94% yield.

2. H, ^{19}F, and ^{13}C-NMR spectra of 0F–4F

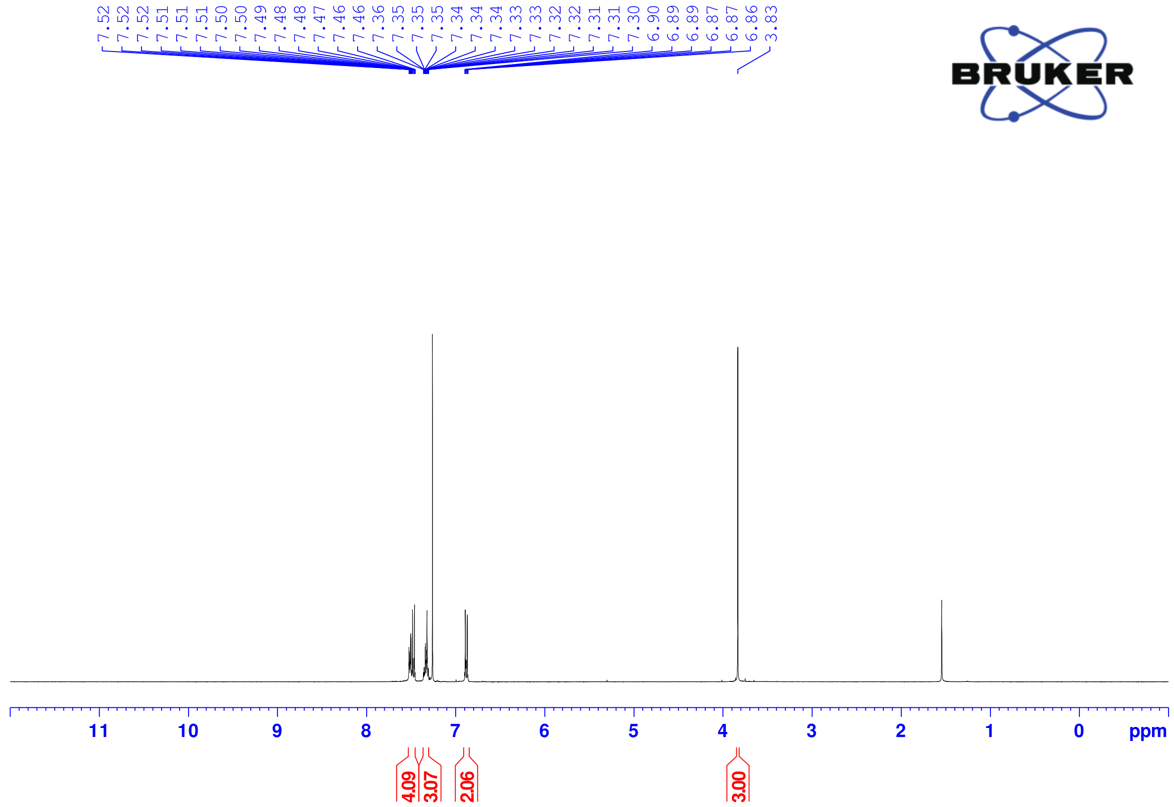

Figure S2. ^{1}H-NMR spectrum of **0F.**

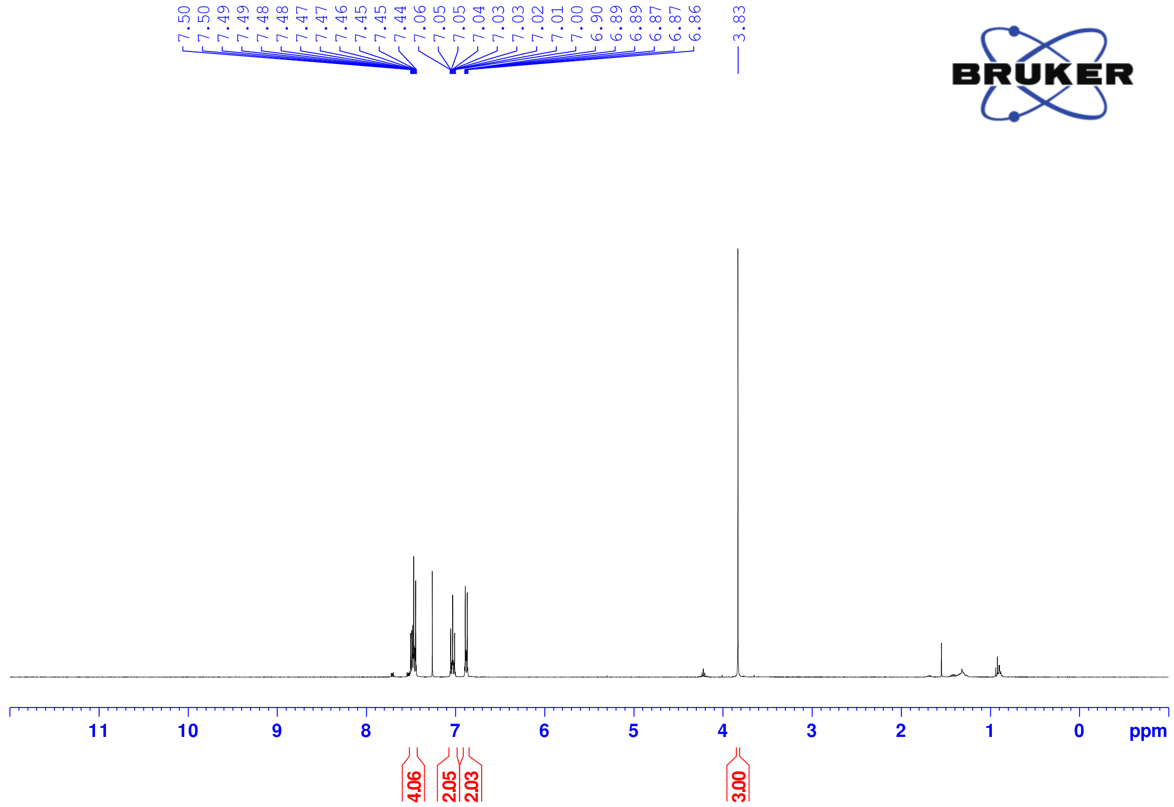

Figure S3. ^{1}H-NMR spectrum of **1F.**

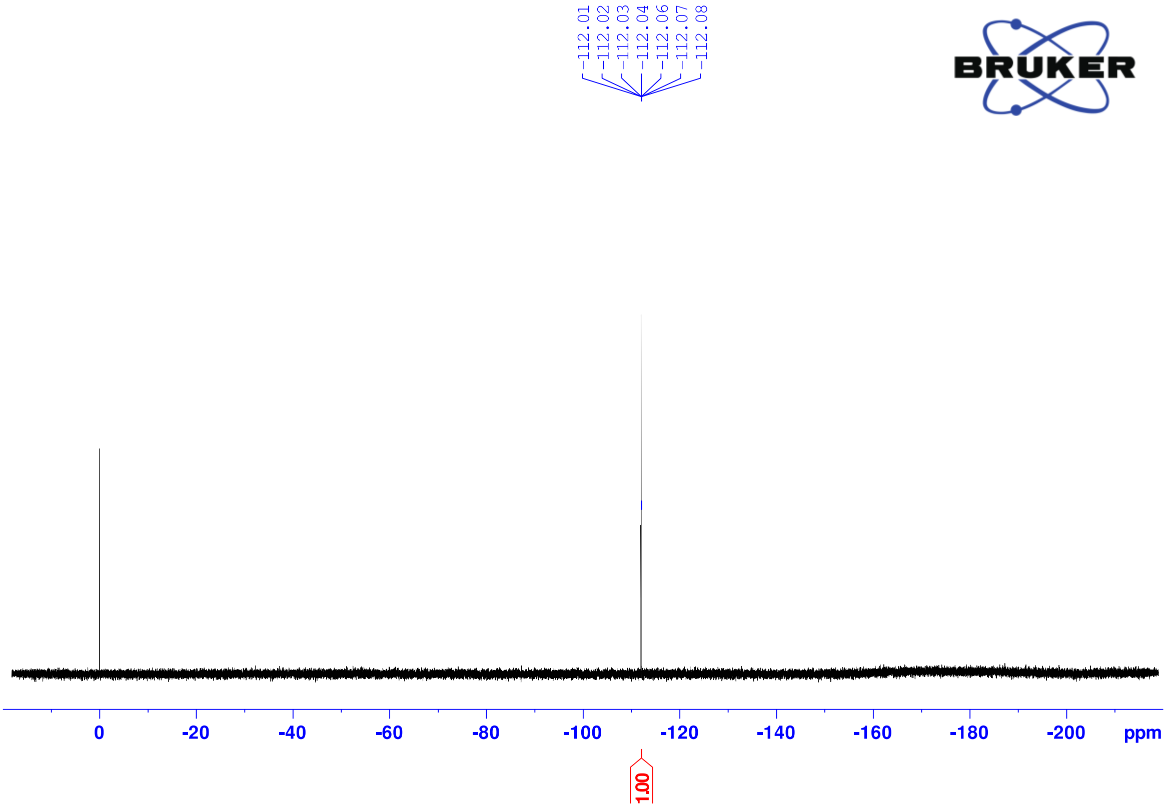

Figure S4. ^{19}F-NMR spectrum of **1F.**

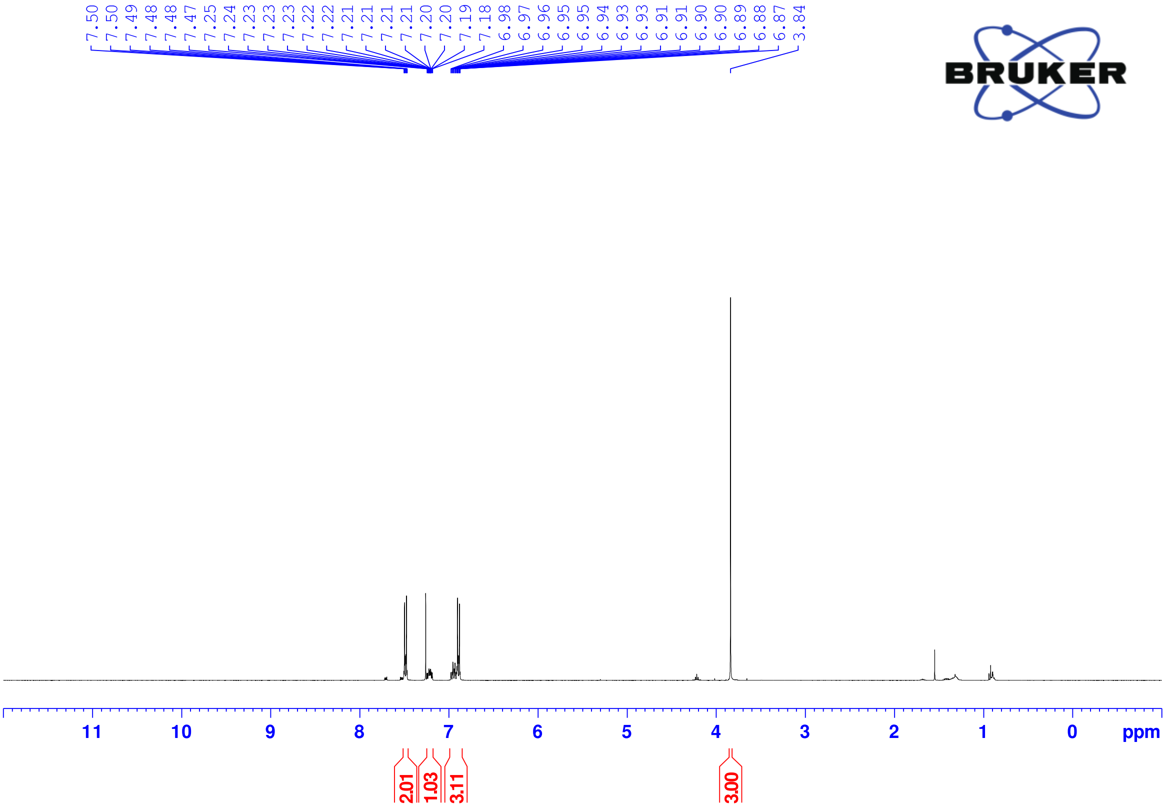

Figure S5. ^{1}H-NMR spectrum of **3Fa.**

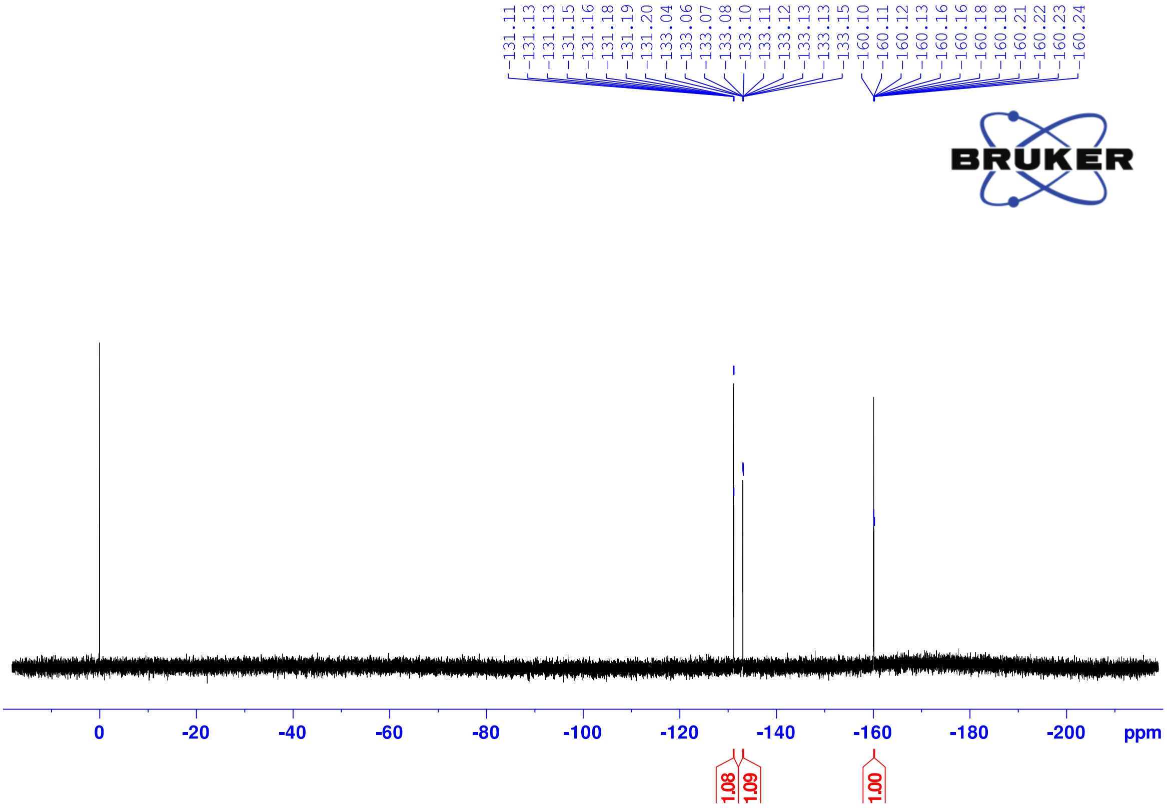

Figure S6. ^{19}F-NMR spectrum of **3Fa.**

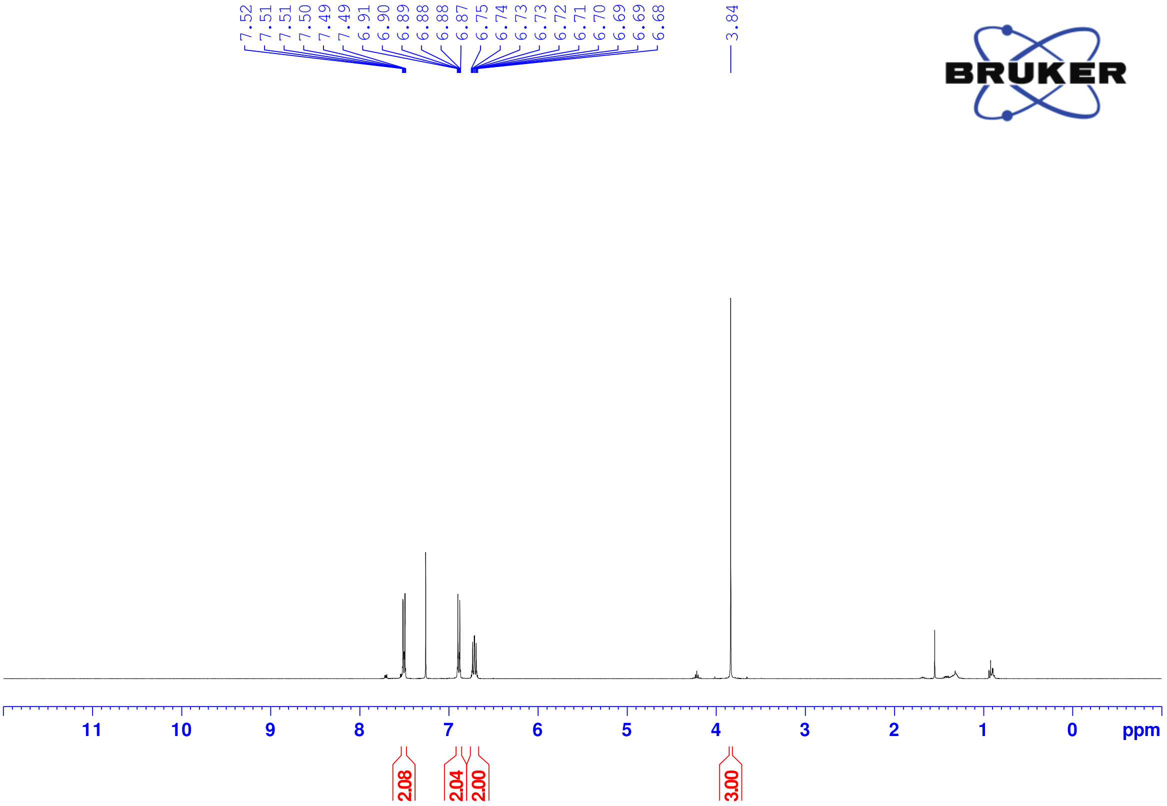

Figure S7. ^{1}H-NMR spectrum of **3Fb.**

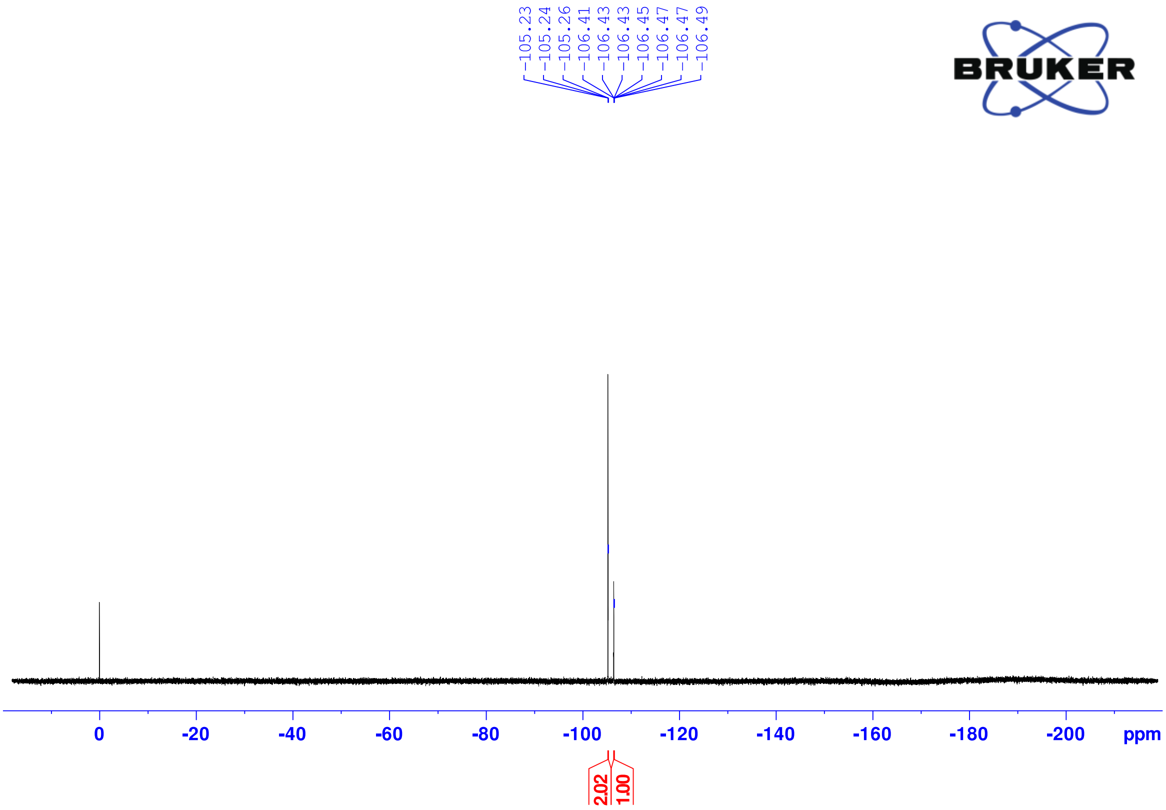

Figure S8. ^{19}F-NMR spectrum of **3Fb.**

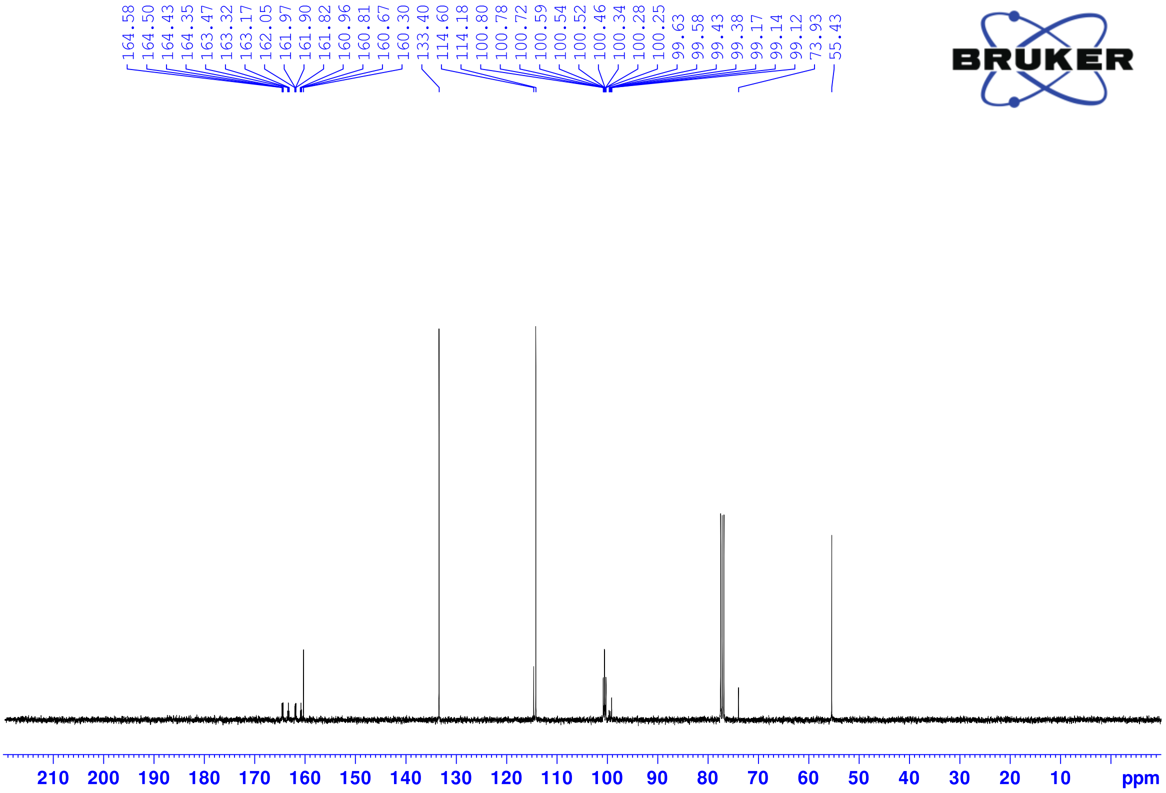

Figure S9. ^{13}C-NMR spectrum of **3Fb.**

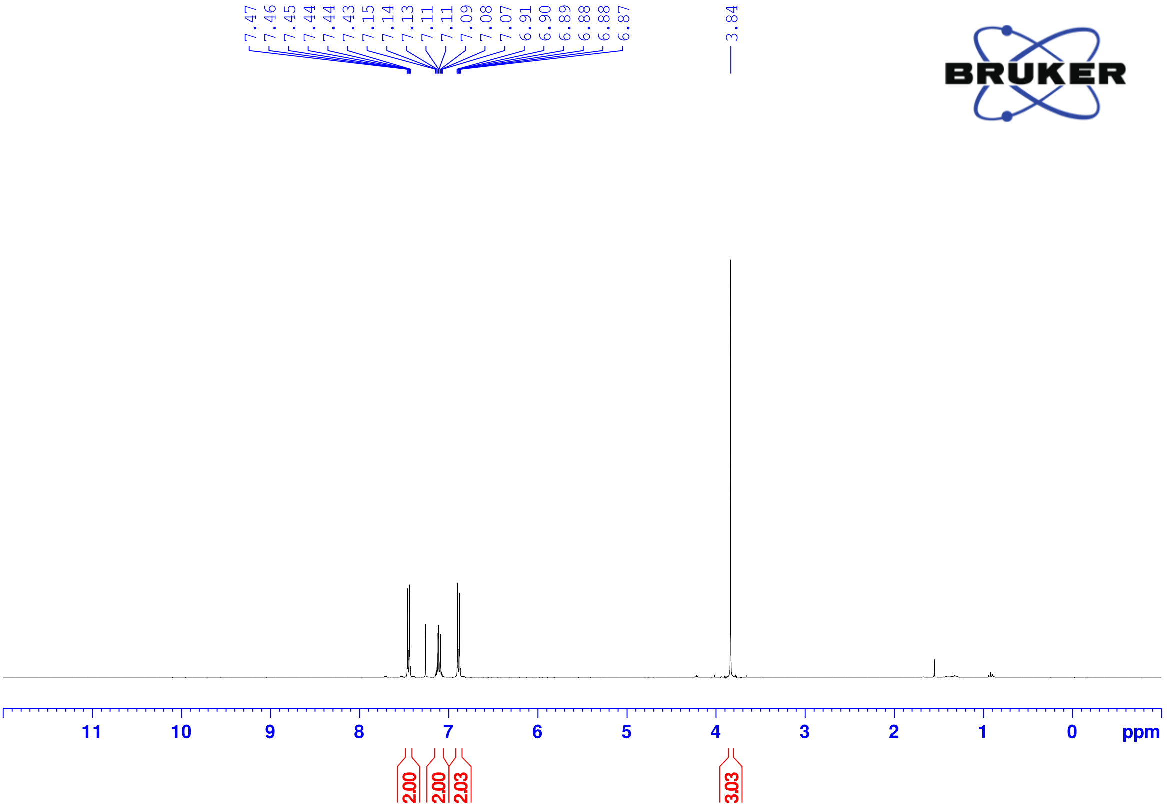

Figure S10. ^{1}H NMR spectrum of **3Fc.**

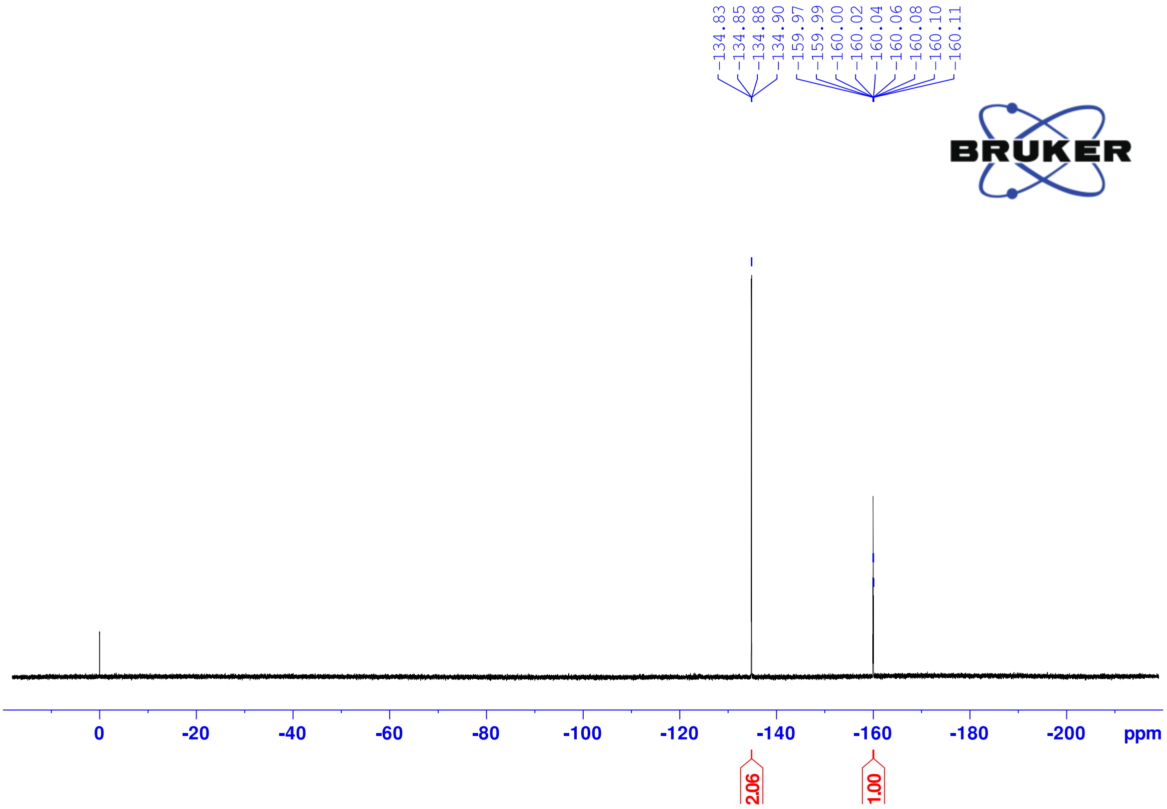

Figure S11. ^{19}F-NMR spectrum of **3Fc.**

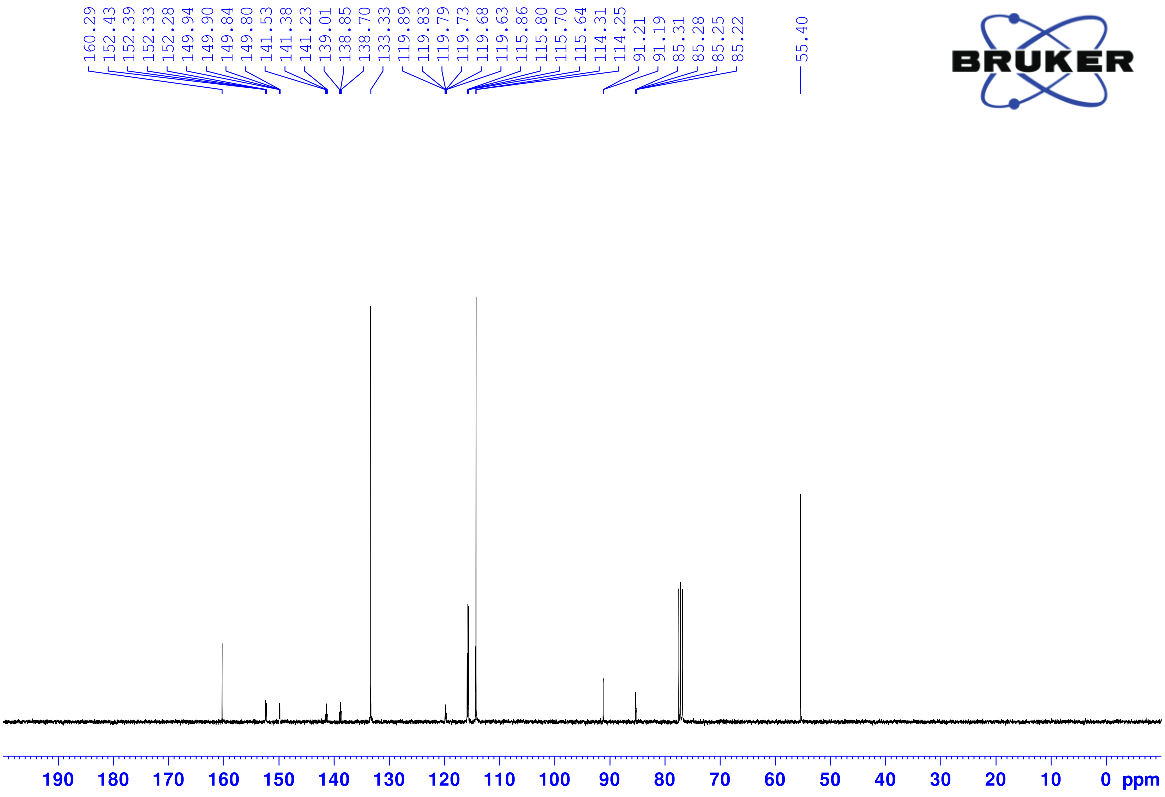

Figure S12. ^{13}C-NMR spectrum of **3Fc.**

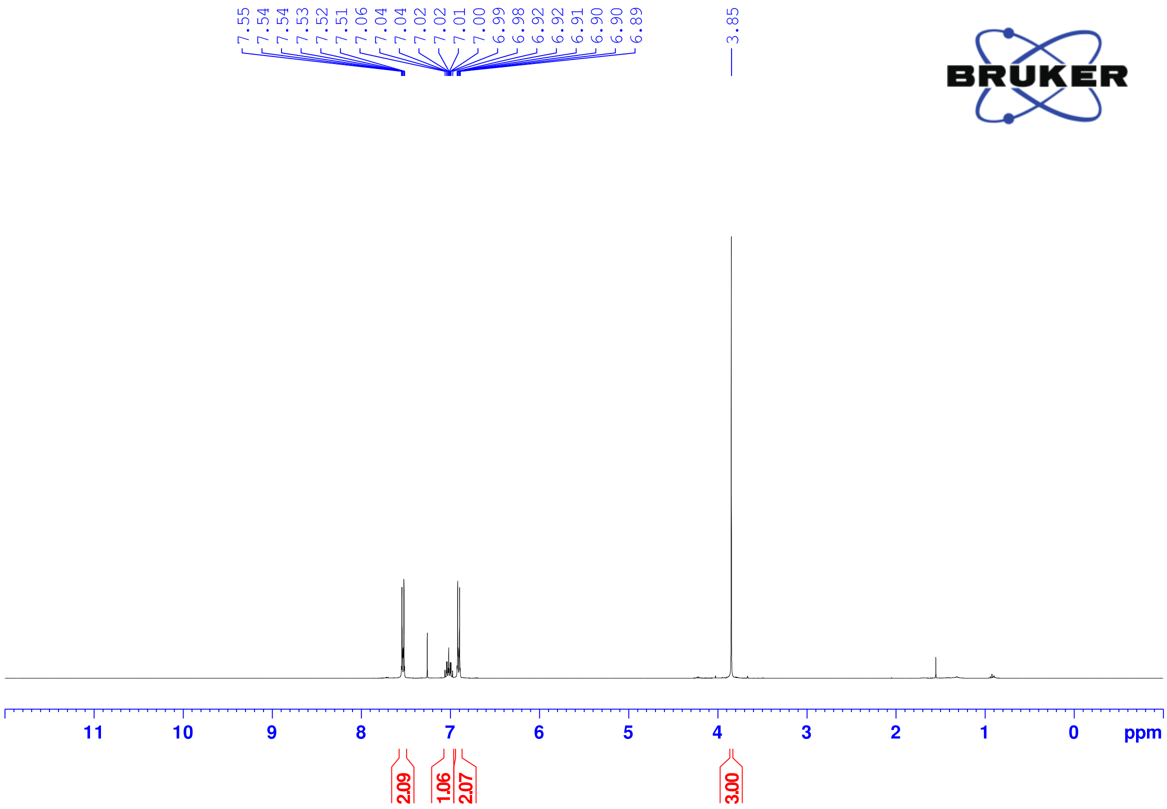

Figure S13. ^{1}H-NMR spectrum of **4F.**

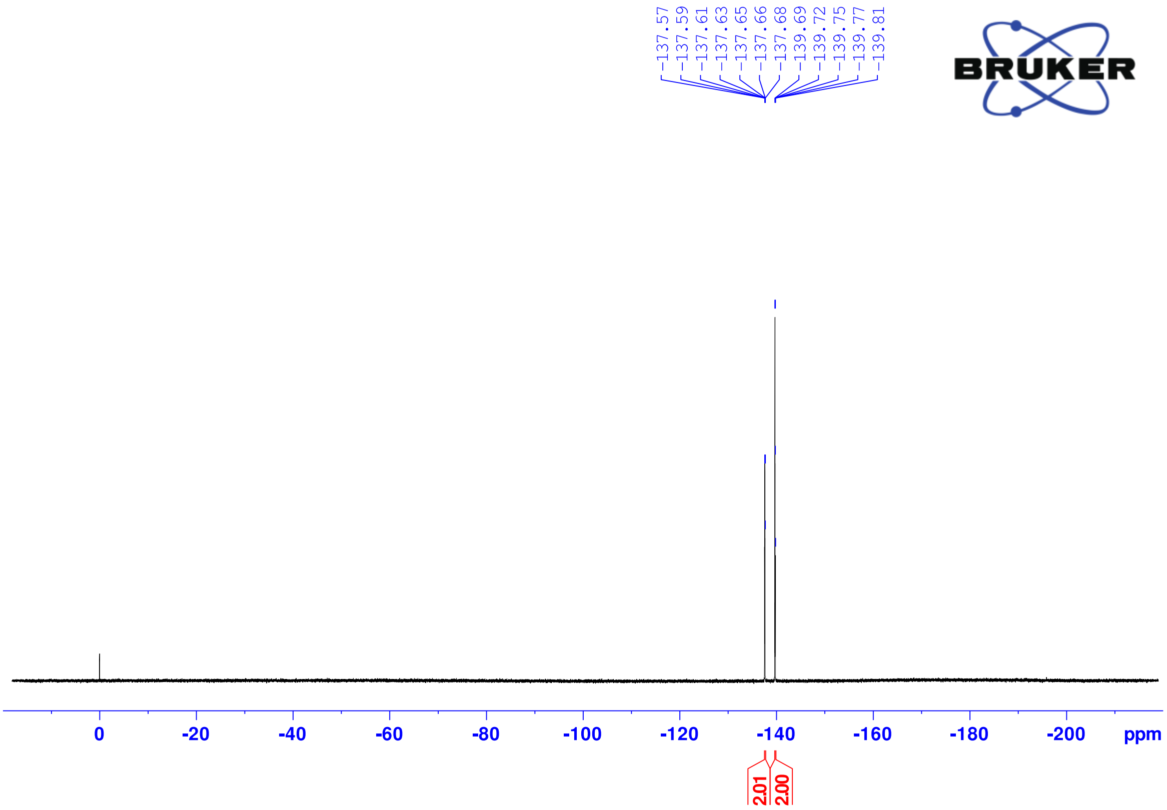

Figure S14. ^{19}F-NMR spectrum of **4F.**

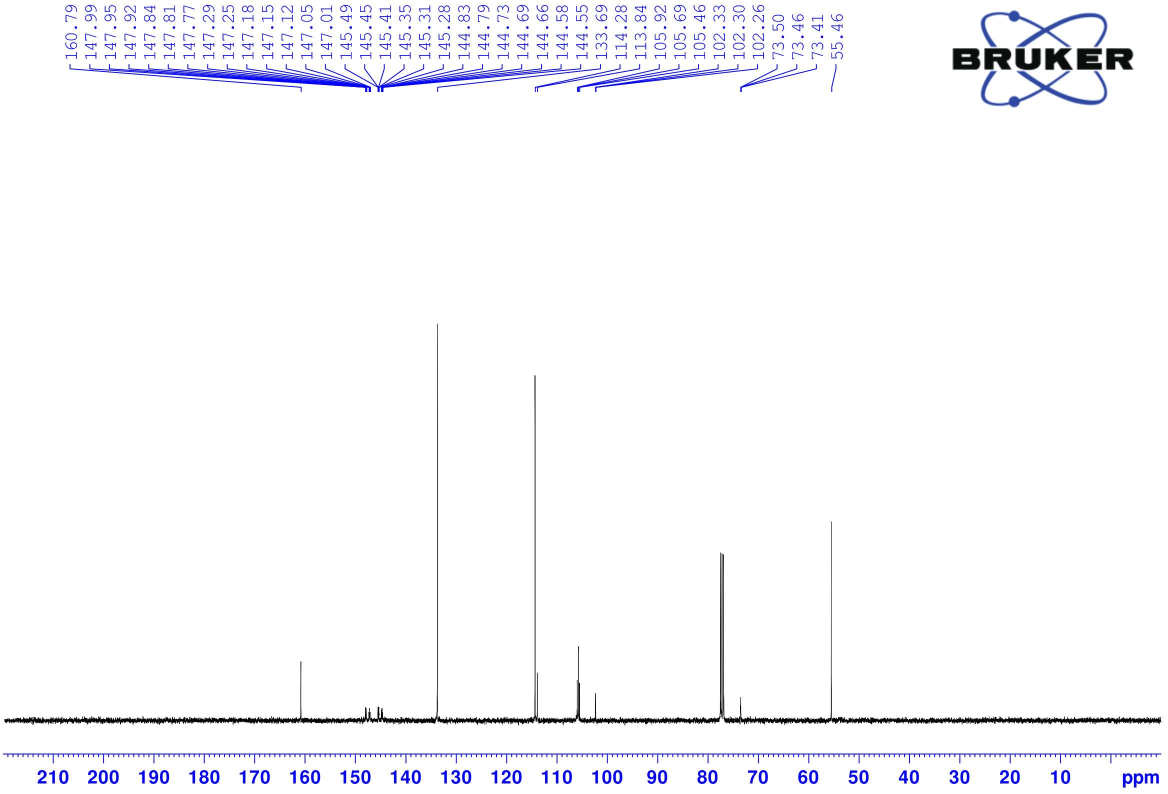

Figure S15. ^{13}C-NMR spectrum of **4F.**

3. Absorption and PL spectra of THF solution

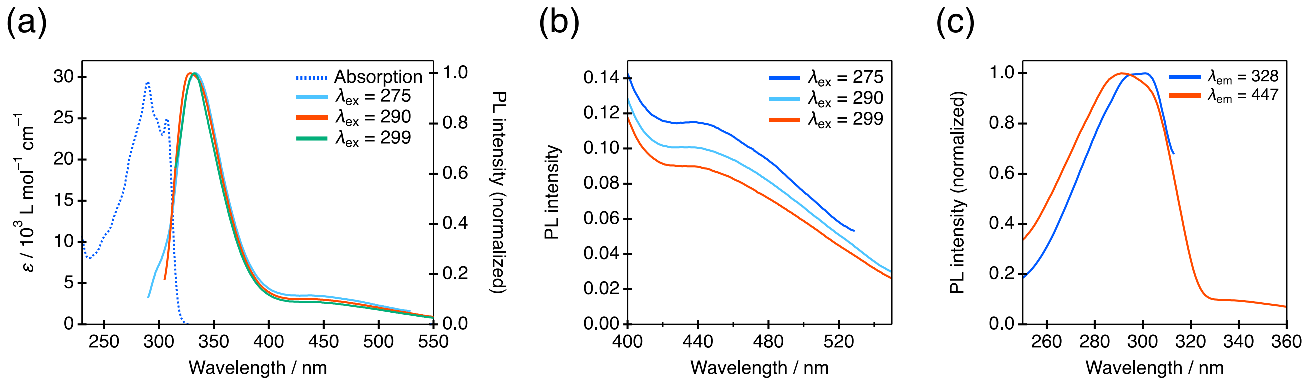

Figure S16. (**a**) Absorption and emission spectra, (**b**) excitation spectra, (**c**) expansion of long-wavelength region of emission spectra of **0F** in 10^{-5} mol L^{-1} THF solution.

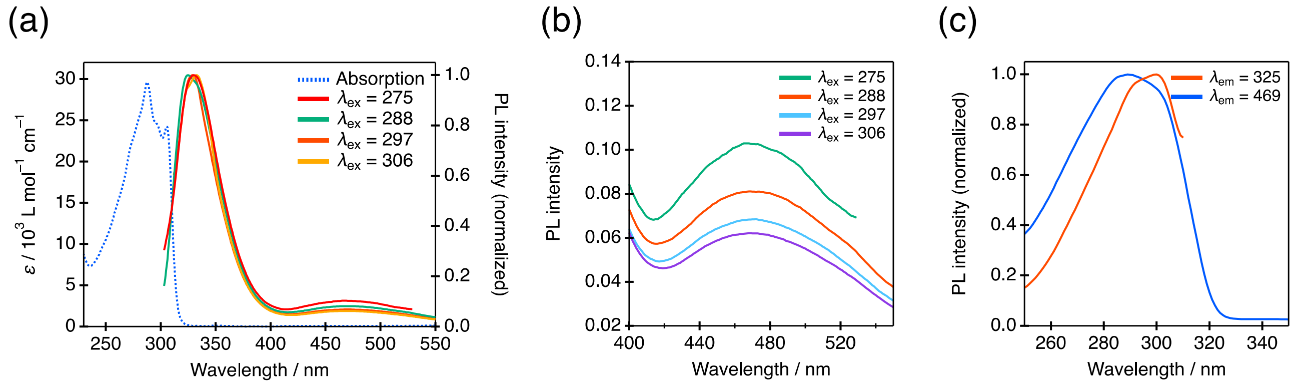

Figure S17. (**a**) Absorption and emission spectra, (**b**) excitation spectra, (**c**) expansion of long-wavelength region of emission spectra of **1F** in 10^{-5} mol L^{-1} THF solution.

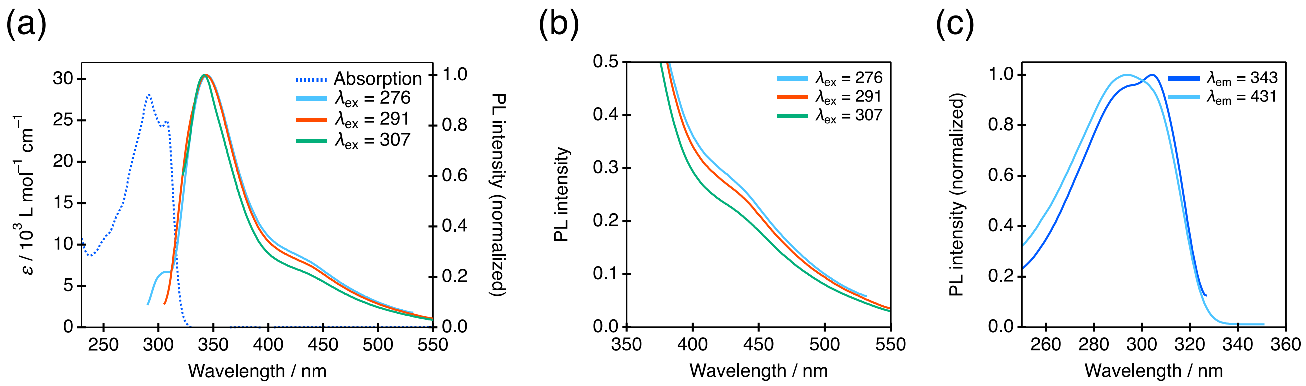

Figure S18. (**a**) Absorption and emission spectra, (**b**) excitation spectra, (**c**) expansion of long-wavelength region of emission spectra of **3Fa** in 10^{-5} mol L^{-1} THF solution.

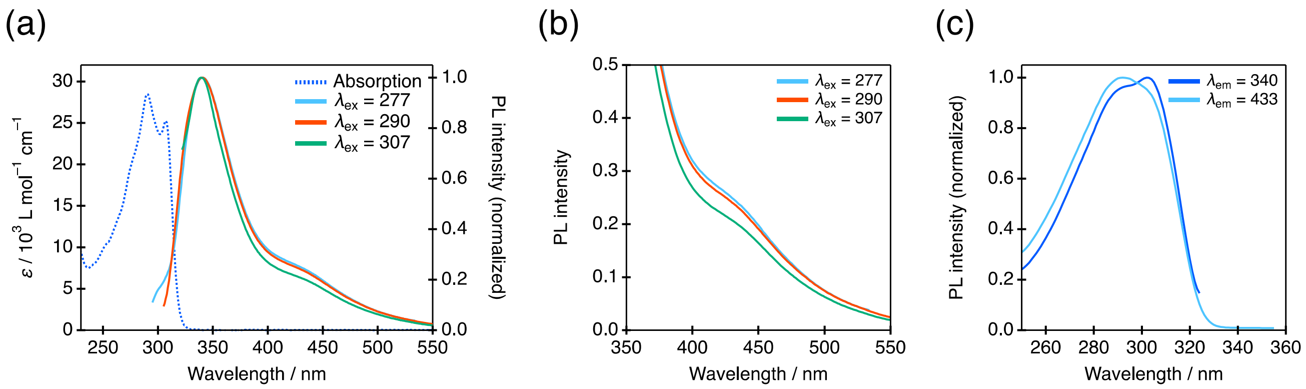

Figure S19. (**a**) Absorption and emission spectra, (**b**) excitation spectra, (**c**) expansion of long-wavelength region of emission spectra of **3Fb** in 10^{-5} mol L^{-1} THF solution.

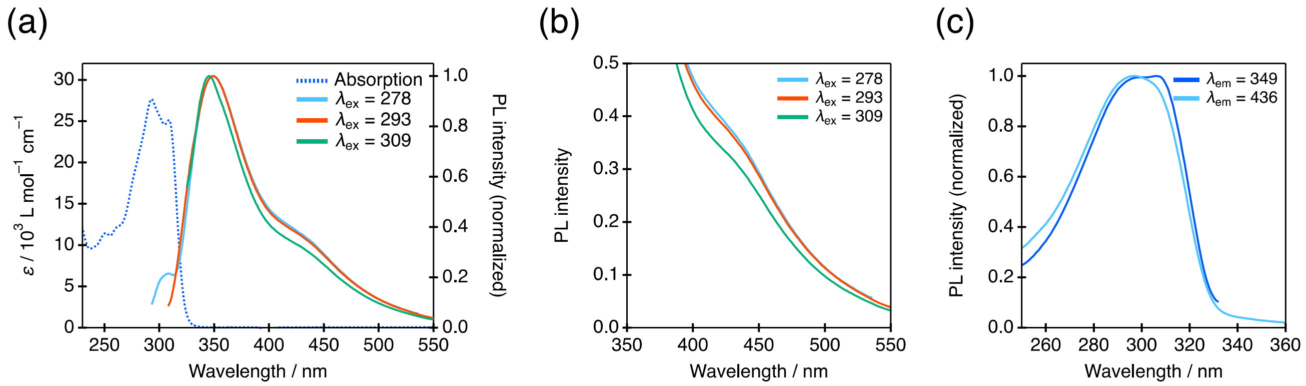

Figure S20. (**a**) Absorption and emission spectra, (**b**) excitation spectra, (**c**) expansion of long-wavelength region of emission spectra of **3Fc** in 10^{-5} mol L^{-1} THF solution.

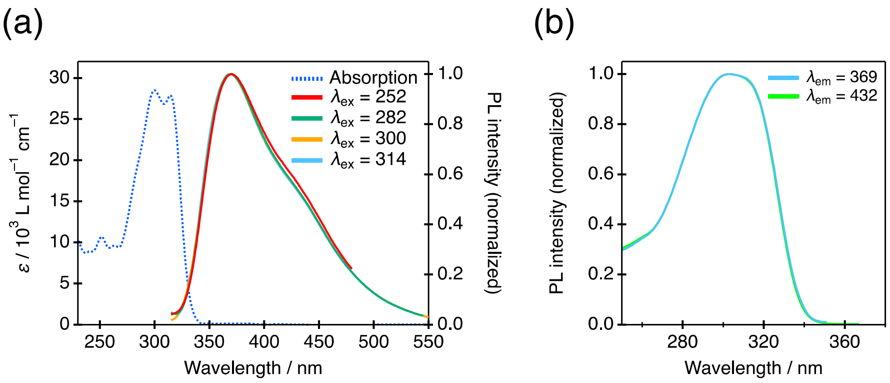

Figure S21. (**a**) Absorption and emission spectra, (**b**) excitation spectra of **4F** in 10^{-5} mol L^{-1} THF solution.

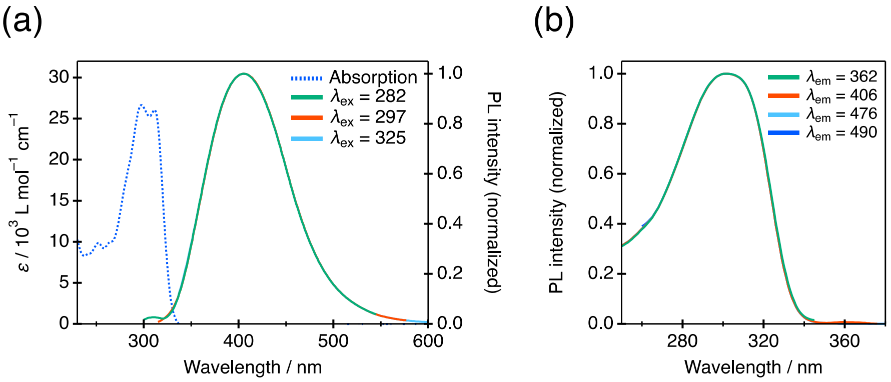

Figure S22. (**a**) Absorption and emission spectra, (**b**) excitation spectra of **5F** in 10^{-5} mol L^{-1} THF solution.

Table S1. Stokes Shift of **0F–5F** in THF solution.

Compound	λ_{abs}/nm	λ_{PL}/nm	Stokes Shift ($\Delta\lambda$) /nm
**0F**	290	328	38
**1F**	288	330	42
**3Fa**	291	343	52
**3Fb**	290	340	50
**3Fc**	293	349	56
**4F**	300	369	69
**5F**	297	406	109

4. Absorption and PL Spectra in Hexane and Dichloromethane

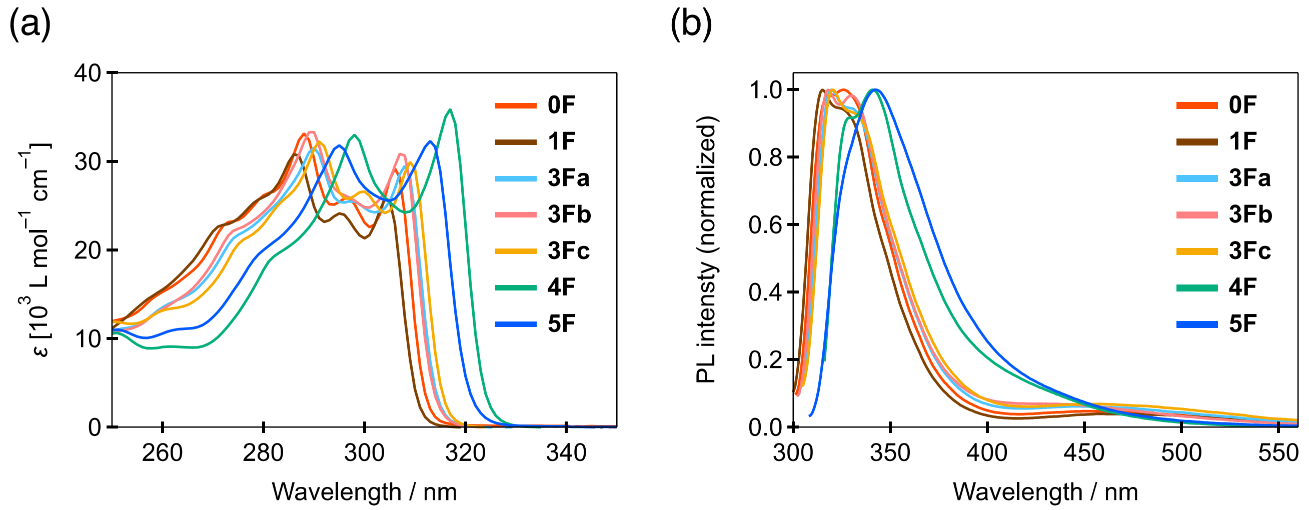

Figure S23. (**a**) UV-vis spectra and, (**b**) emission spectra of **0F–5F** excited at maximum absorption wavelength in 10^{-5} mol L^{-1} hexane solution.

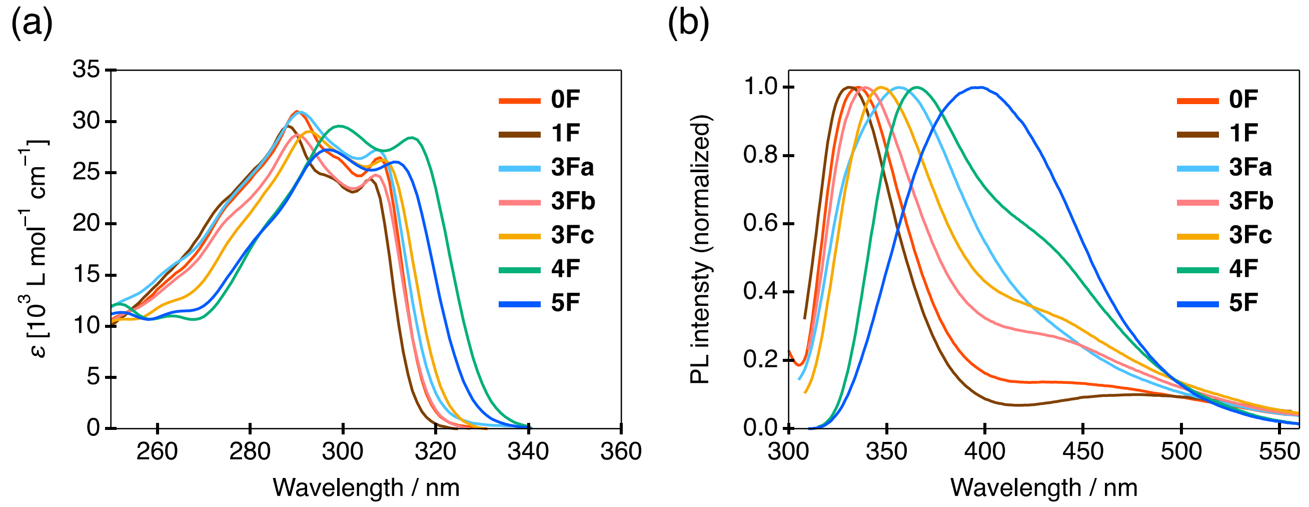

Figure S24. (**a**) UV-vis spectra and (**b**) emission spectra of **0F–5F** excited at maximum absorption wavelength in 10^{-5} mol L^{-1} dichloromethane solution.

5. Solvatochromic PL Properties of 3Fa–c

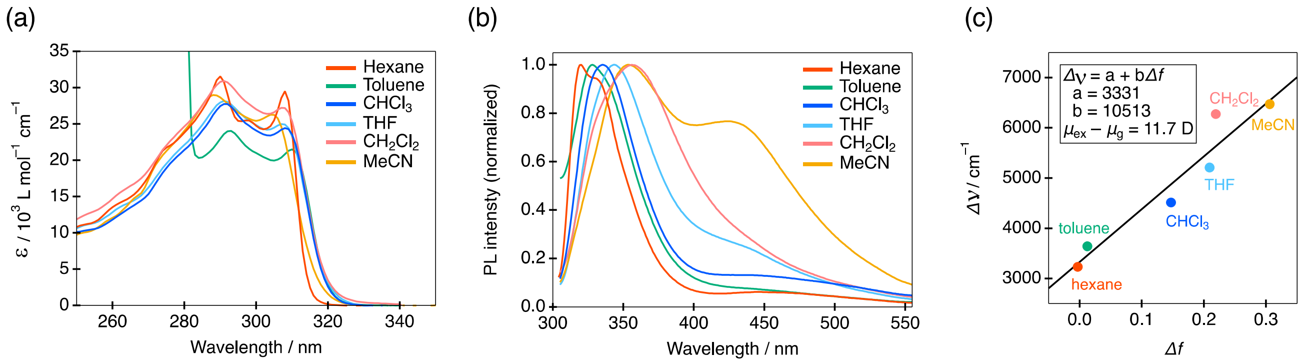

Figure S25. (**a**) Absorption spectra, (**b**) emission spectra of **3Fa** excited at maximum absorption wavelength in 10^{-5} mol L^{-1} solution (**c**) Lippert-Mataga plot for **3Fa**.

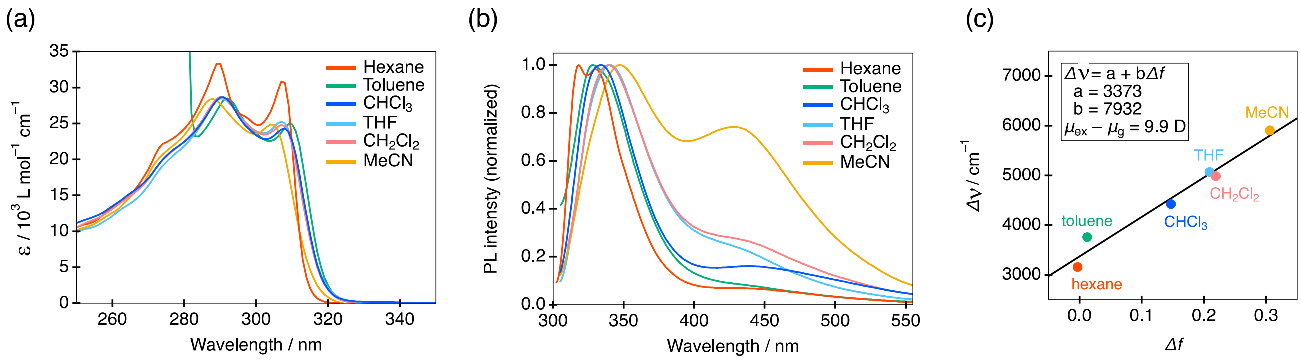

Figure S26. (**a**) Absorption spectra, (**b**) emission spectra of **3Fb** excited at maximum absorption wavelength in 10^{-5} mol L^{-1} solution (**c**) Lippert-Mataga plot for **3Fb**.

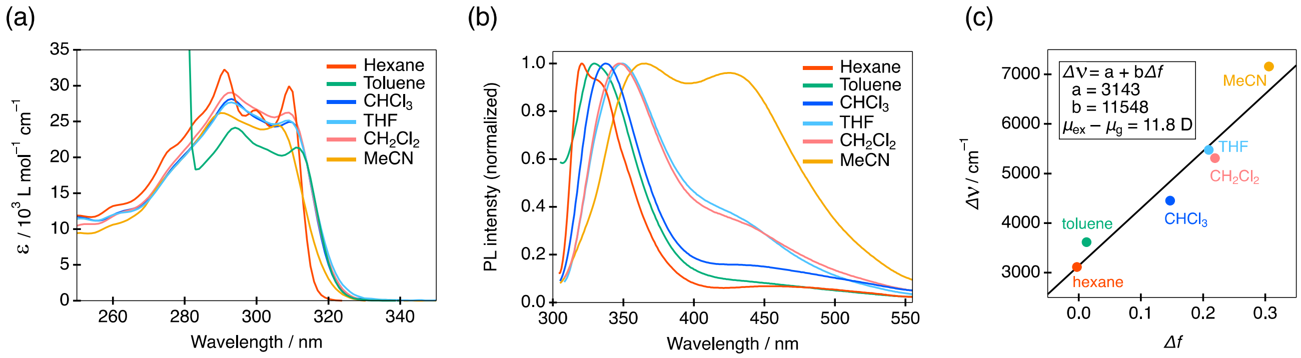

Figure S27. (**a**) Absorption spectra, (**b**) emission spectra of **3Fc** excited at maximum absorption wavelength in 10^{-5} mol L^{-1} solution, (**c**) Lippert-Mataga plot for **3Fc**.

6. PL Spectra of THF/Water Mixed Solution

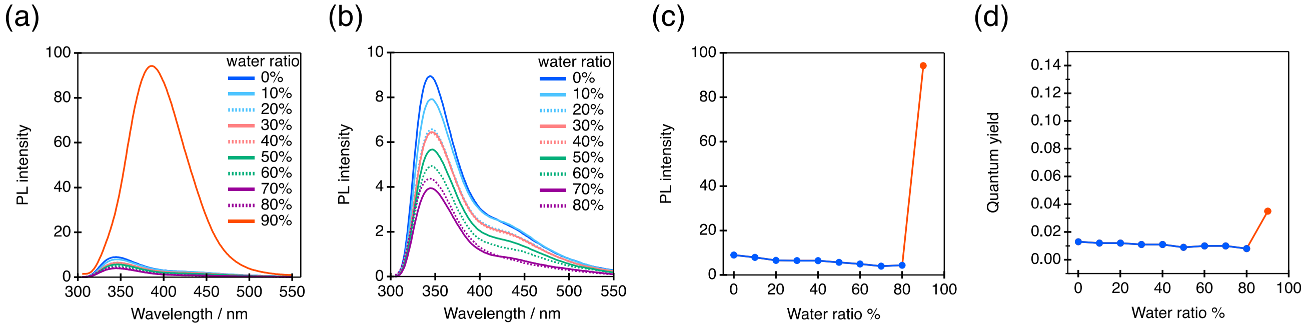

Figure S28. Emission spectra of (**a**) water ratio: 0–90%, (**b**) water ratio: 0–80%, (**c**) Emission intensity respect to water ratio, (**d**) Quantum yield respect to water ratio of **3Fa** in THF/water mixed solution.

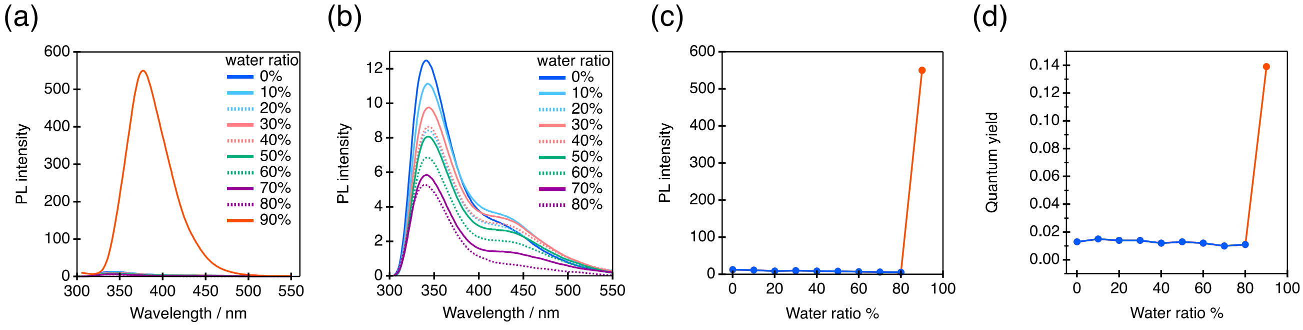

Figure S29. Emission spectra of (**a**) water ratio: 0–90%, (**b**) water ratio: 0–80%, (**c**) Emission intensity respect to water ratio, (**d**) Quantum yield respect to water ratio of **3Fb** in THF/water mixed solution.

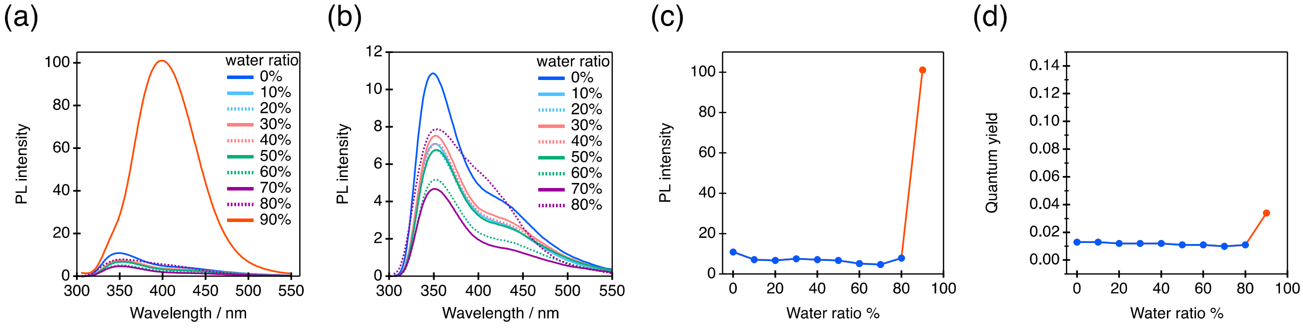

Figure S30. Emission spectra of (**a**) water ratio: 0–90%, (**b**) water ratio: 0–80%, (**c**) Emission intensity respect to water ratio, (**d**) Quantum yield respect to water ratio of **3Fc** in THF/water mixed solution.

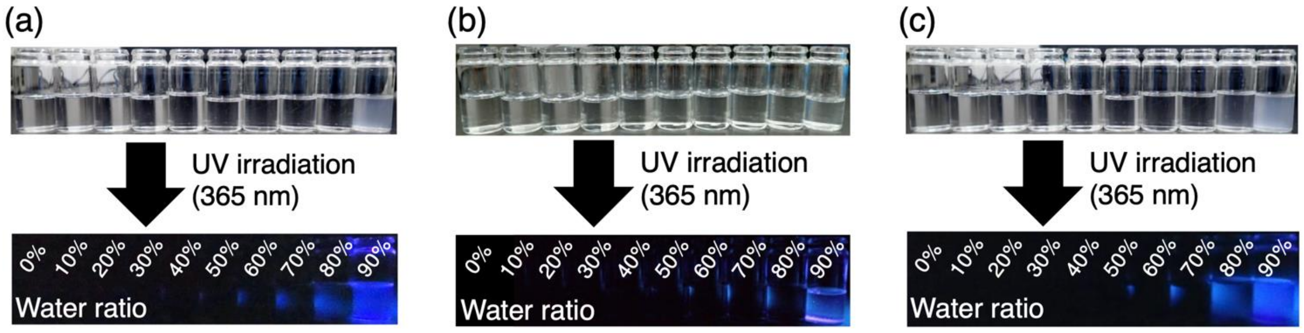

Figure S31. Photographs of THF/water mixed solution (**a**) **3Fa**, (**b**) **3Fb**, and (**c**) **3Fc**.

7. PL Spectra of Crystal

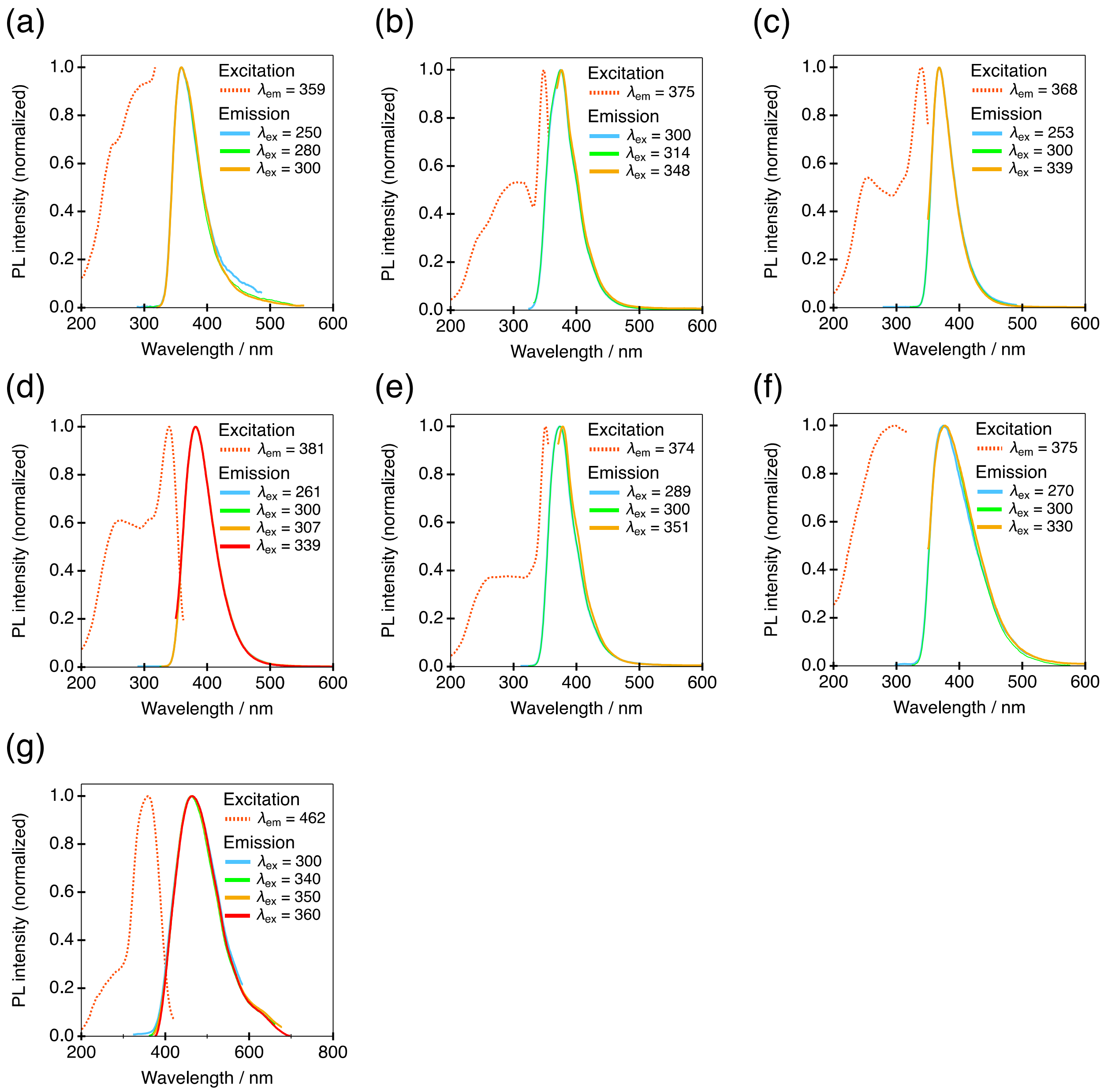

Figure S32. Excitation and emission spectra in crystalline state of (**a**) **0F**, (**b**) **1F**, (**c**) **3Fa** (**d**) **3Fb**, (**e**) **3Fc**, (**f**) **4F**, (**g**) **5F**.

8. PL Lifetime of Solution and Crystal

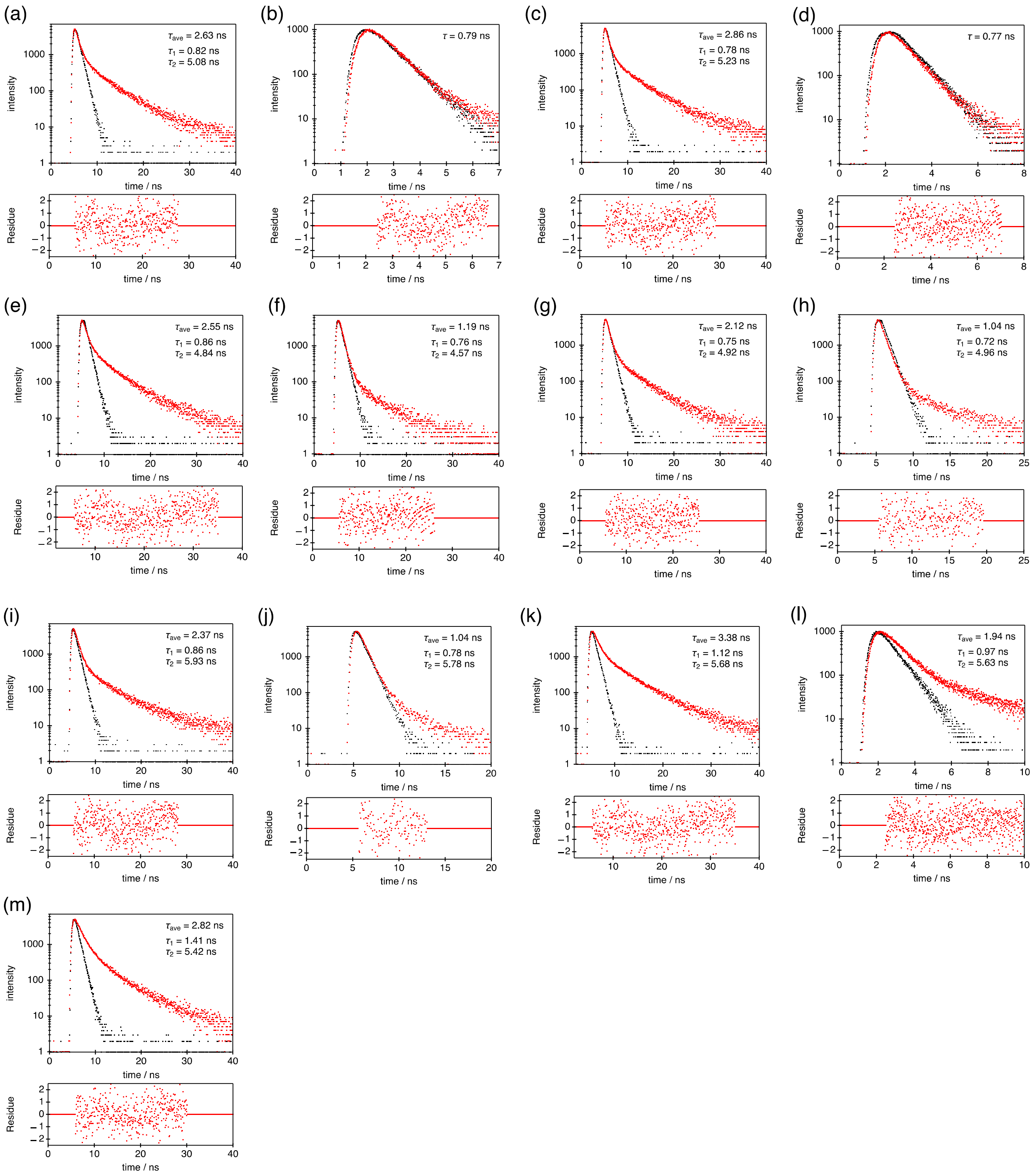

Figure S33. PL lifetime measurement in 10^{-5} mol L^{-1} THF solution of (**a**) **0F** (λ_{em} = 328 nm), (**b**) **0F** (λ_{em} = 447 nm), (**c**) **1F** (λ_{em} = 330 nm), (**d**) **1F** (λ_{em} = 469 nm), (**e**) **3Fa** (λ_{em} = 343 nm), (**f**) **3Fa** (λ_{em} = 431 nm), (**g**) **3Fb** (λ_{em} = 340 nm), (**h**) **3Fb** (λ_{em} = 433 nm), (**i**) **3Fc** (λ_{em} = 349 nm), (**j**) **3Fc** (λ_{em} = 436 nm), (**k**) **4F** (λ_{em} = 369 nm), (**l**) **4F** (λ_{em} = 432 nm), (**m**) **5F** (λ_{em} = 406 nm). Black plots showed Instrument Response Function (IRF).

Table S2. PL lifetime in 10^{-5} mol L^{-1} THF solution.

Compound	λ_{max}/nm	τ_{avg}/ns	τ_1/ns	τ_2/ns
**0F**	328	2.63	0.82	5.08
	447	0.79	-	-
**1F**	330	2.86	0.78	5.23
	469	0.77	-	-
**3Fa**	343	2.55	0.86	4.84
	431	1.19	0.76	4.57
**3Fb**	340	2.12	0.75	4.92
	433	1.04	0.72	4.96
**3Fc**	349	2.37	0.86	5.93
	436	1.04	0.78	5.78
**4F**	369	3.38	1.12	5.68
	432	1.94	0.97	5.63
**5F**	406	2.82	1.41	5.42

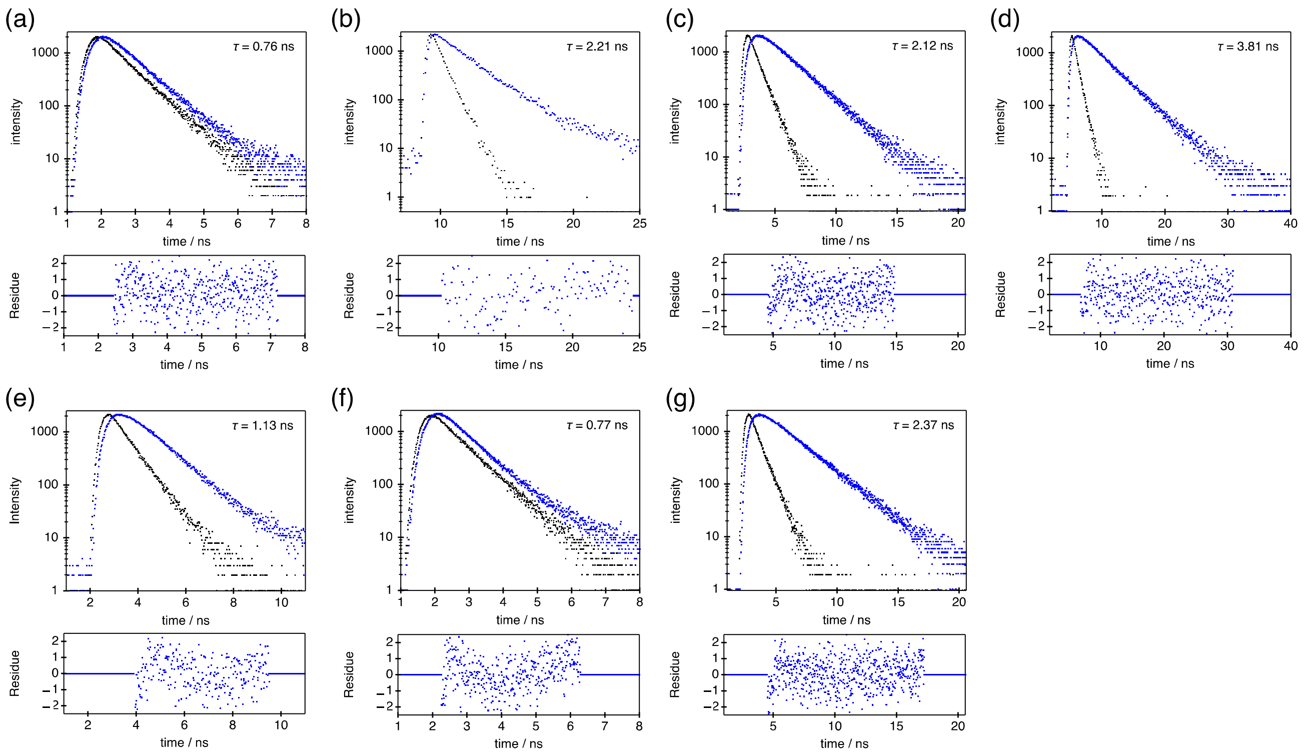

Figure S34. PL lifetime measurement in crystal monitored at maximum emission wavelength of (**a**) **0F**, (**b**) **1F**, (**c**) **3Fa**, (**d**) **3Fb**, (**e**) **3Fc**, (**f**) **4F**, (**g**) **5F**. Black plots showed Instrument Response Function (IRF).

9. Cyclic Voltammetry (CV)

Table S3. Electrical properties of **0F**–**5F**.

Compound	E_{Ox}/V	E_{Red}/V	E_{HOMO}/eV	E_{LUMO}/eV	ΔE/eV
**0F**	1.01	−2.71	−5.81	−2.09	3.72
**1F**	1.01	−2.74	−5.81	−2.06	3.75
**3Fa**	1.16	−2.47	−5.96	−2.33	3.63
**3Fb**	1.16	−2.53	−5.96	−2.27	3.69
**3Fc**	1.16	−2.42	−5.96	−2.38	3.58
**4F**	1.27	−2.30	−6.07	−2.50	3.57
**5F**	1.22	−2.19	−6.02	−2.61	3.41

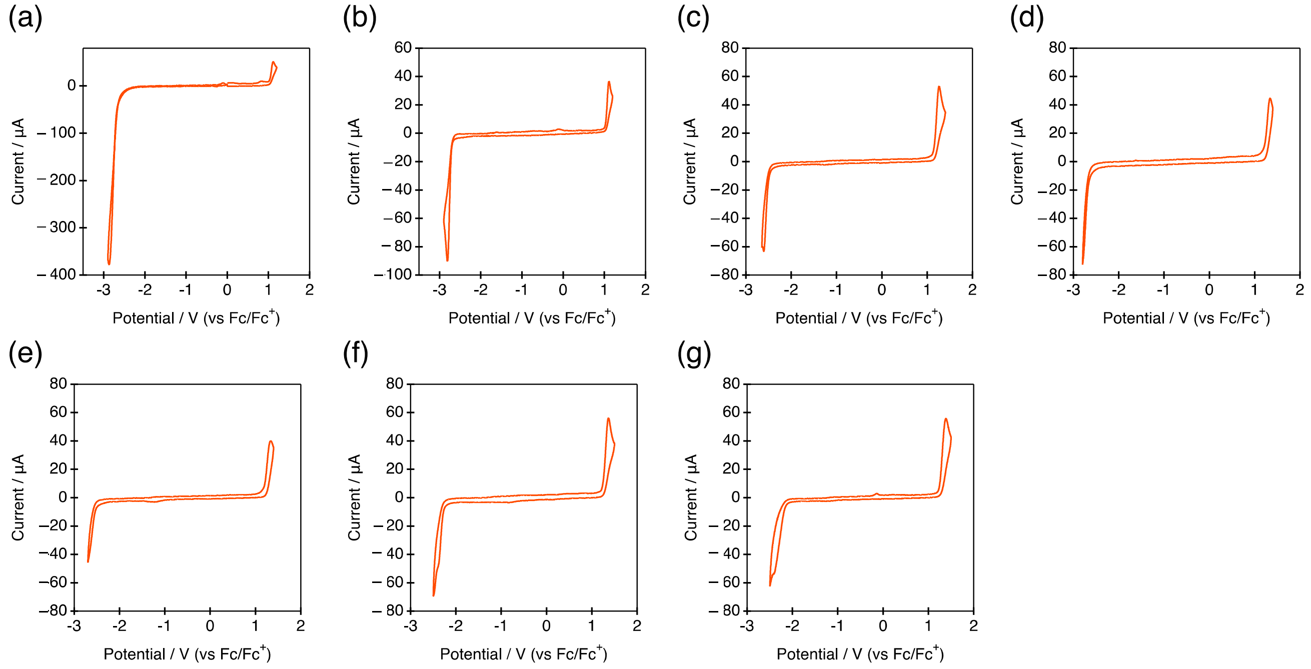

Figure S35. Cyclic voltammograms of (**a**) **0F**, (**b**) **1F**, (**c**) **3Fa**, (**d**) **3Fb**, (**e**) **3Fc**, (**f**) **4F**, (**g**) **5F**.

10. Crystallographic data

Table S4. Crystallographic data of **3Fa**, **3Fb**, and **3Fc**.

	**3Fa**	**3Fb**	**3Fc**
CCDC #	2070935	2070936	2070937
Empirical Formula	$C_{15}H_9F_3O$	$C_{15}H_9F_3O$	$C_{15}H_9F_3O$
Formula weight	262.22	262.22	262.22
Temperature [K]	293	293	293
Crystal Color / Habit	Colorless / Block	Colorless / Block	Colorless / Block
Crystal Size [mm]	0.66 × 0.575 × 0.321	0.811 × 0.48 × 0.205	0.33 × 0.243 × 0.138
Crystal System	orthorhombic	triclinic	monoclinic
Space Group	*Pnma*	*P*1	$P2_1/n$
a [Å]	14.1431(8)	8.0147(6)	7.3569(5)
b [Å]	6.6476(4)	11.8893(5)	15.2159(8)
c [Å]	13.1294(6)	12.9981(3)	11.2739(6)
α [°]	90	85.584(3)	90
β [°]	90	83.905(4)	100.539(6)
γ [°]	90	89.238(4)	90
V [Å^3]	1234.40(12)	1227.90(11)	1240.73(13)
Z	4	4	4
R [$F^2 > 2\sigma(F^2)$] [a]	0.0504	0.0621	0.0467
$wR2$ (F^2) [b]	0.1752	0.1990	0.1404

[a] $R = \Sigma||F_o| - |F_c|| / \Sigma|F_o|$. [b] $wR = \{[\Sigma w(|F_o| - |F_c|)]/\Sigma w|F_o|\}^{1/2}$.

Table S5. Crystallographic data of **4F** and **5F**.

	**4F**	**5F**
CCDC #	2070938	2070939
Empirical Formula	$C_{15}H_8F_4O$	$C_{15}H_7F_5O$
Formula weight	280.21	298.21
Temperature [K]	293	293
Crystal Color / Habit	Colorless / Block	Colorless / Prism
Crystal Size [mm]	0.627 × 0.35 × 0.097	0.92 × 0.79 × 0.55
Crystal System	triclinic	monoclinic
Space Group	$P1$	$P2_1/c$
a [Å]	7.5740(6)	11.0681(13)
b [Å]	8.7931(7)	7.6313(8)
c [Å]	9.6293(9)	15.4043(19)
α [°]	93.724(7)	90
β [°]	101.996(7)	103.576(3)
γ[°]	98.902(7)	90
V [Å^3]	616.60(9)	1264.8(3)
Z	2	4
R [$F^2 > 2\sigma(F^2)$] [a]	0.0579	0.0522
$wR2$ (F^2) [b]	0.1889	0.1614

[a] $R = \Sigma||F_o| - |F_c|| / \Sigma|F_o|$. [b] $wR = \{[\Sigma w(|F_o| - |F_c|)]/\Sigma w|F_o|\}^{1/2}$.

11. DFT calculation

Table S6. DFT calculation.

**Compound**	**$\mu_{||}$/debye[a]**	**HOMO/eV**	**LUMO/eV**	***ΔE*/eV**
**0F**	1.1607	−7.0826	−0.7072	6.3754
**1F**	3.1917	−7.0872	−0.6797	6.4075
**3Fa**	4.2798	−7.2399	−0.9042	6.3357
**3Fb**	1.9886	−7.2221	−0.8754	6.3467
**3Fc**	5.8519	−7.2565	−0.9521	6.3044
**4F**	2.8011	−7.3716	−1.2017	6.1699
**5F**	4.6676	−7.3713	−1.1551	6.2162

[a] dipole moment in long molecular axis.

Geometry Optimization of **0F**

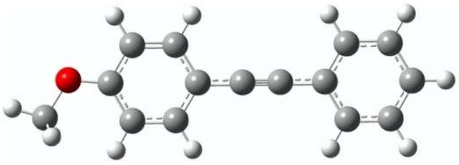

Center Number	Atomic Number	Atomic Type	Coordinates (Angstroms) X	Y	Z
1	6	0	-3.113520	1.419114	−0.000070
2	6	0	−1.730770	1.364414	−0.000061
3	6	0	−1.061938	0.125264	0.000002
4	6	0	−1.825407	−1.048441	0.000055
5	6	0	−3.218189	−1.002937	0.000047
6	6	0	−3.866894	0.235974	−0.000015
7	1	0	−3.636354	2.370439	−0.000119
8	1	0	−1.152395	2.283280	−0.000104
9	1	0	−1.323501	−2.011297	0.000104
10	1	0	−3.777749	−1.930903	0.000090
11	6	0	0.369092	0.068645	0.000009
12	6	0	1.582396	0.023737	0.000014
13	6	0	3.015246	−0.026354	0.000010
14	6	0	3.765677	1.161081	0.000074
15	6	0	3.683173	−1.262069	−0.000062
16	6	0	5.156465	1.109368	0.000068
17	1	0	3.250118	2.116688	0.000129
18	6	0	5.074354	−1.304842	−0.000068
19	1	0	3.103758	−2.180368	−0.000113
20	6	0	5.814747	−0.121551	−0.000003
21	1	0	5.728066	2.032626	0.000118
22	1	0	5.581722	−2.264918	−0.000124
23	1	0	6.899931	−0.158415	−0.000008
24	8	0	−5.213811	0.392563	−0.000029
25	6	0	−6.018743	−0.776644	0.000023
26	1	0	−5.833294	−1.378707	0.895950
27	1	0	−7.050660	−0.428695	0.000001
28	1	0	−5.833284	−1.378793	−0.895844

Dipole moment (field-independent basis, Debye):

X = −1.1607 Y = −1.6619 Z = 0.0001 Tot = 2.0271

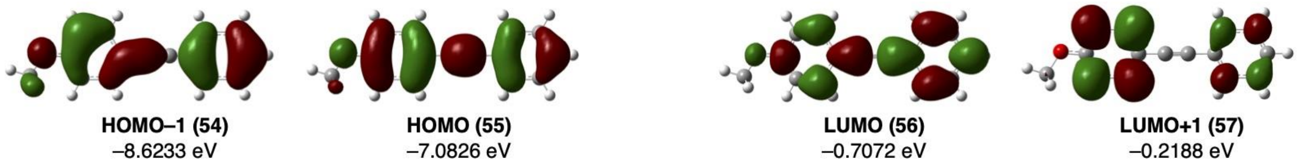

Excited State 1: Singlet-A 4.3321 eV 286.20 nm f = 1.3159 < S**2 > = 0.000

55 -> 56 0.68941

Geometry Optimization of **1F**

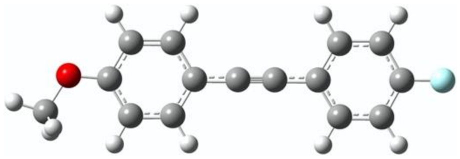

Center Number	Atomic Number	Atomic Type	Coordinates (Angstroms) X	Y	Z
1	6	0	−4.669015	1.150074	−0.000188
2	6	0	−3.278737	1.188261	−0.000139
3	6	0	−2.530648	−0.000795	−0.000089
4	6	0	−3.202425	−1.234590	−0.000089
5	6	0	−4.592364	−1.283857	−0.000139
6	6	0	−5.294554	−0.087848	−0.000187
7	1	0	−5.263318	2.057525	−0.000227
8	1	0	−2.764287	2.143842	−0.000139
9	1	0	−2.628691	−2.155804	−0.000051
10	1	0	−5.128201	−2.227012	−0.000140
11	6	0	−1.098326	0.044836	−0.000040
12	6	0	0.114935	0.084067	0.000002
13	6	0	1.546264	0.132754	0.000050
14	6	0	2.302598	−1.045514	0.000185
15	6	0	2.222149	1.368003	−0.000040
16	6	0	3.695596	−1.008169	0.000231
17	1	0	1.795087	−2.005422	0.000257
18	6	0	3.605204	1.414456	0.000004
19	1	0	1.649310	2.290322	−0.000147
20	6	0	4.351598	0.226893	0.000139
21	1	0	4.249643	−1.939434	0.000337
22	1	0	4.133629	2.362676	−0.000066
23	8	0	5.699335	0.375490	0.000173
24	6	0	6.497324	−0.798513	0.000291
25	1	0	6.308291	−1.399545	−0.895571
26	1	0	7.531284	−0.456710	0.000288
27	1	0	6.308247	−1.399395	0.896245
28	9	0	−6.644807	−0.130590	−0.000235

Dipole moment (field-independent basis, Debye):

X = 3.1917 Y = −1.6103 Z = 0.0002 Tot = 3.5749

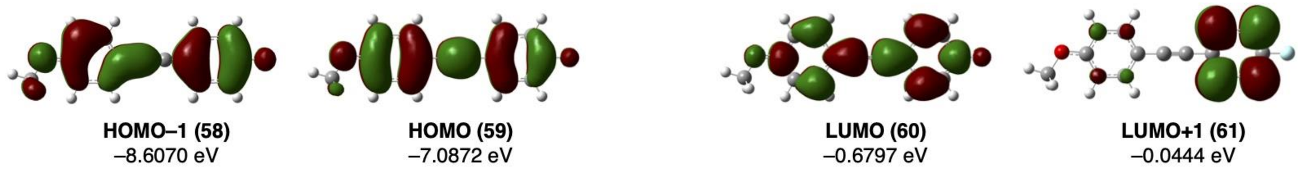

Excited State 1: Singlet-A 4.3587 eV 284.45 nm f = 1.3039 < S**2 > = 0.000

59 -> 60 0.68977

Geometry Optimization of **3Fa**

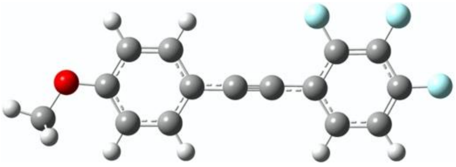

Center Number	Atomic Number	Atomic Type	Coordinates (Angstroms) X	Y	Z
1	6	0	4.249020	−1.032653	0.000003
2	6	0	2.861955	−1.161404	0.000003
3	6	0	2.032007	−0.033764	0.000000
4	6	0	2.623671	1.243924	0.000000
5	6	0	4.000278	1.380342	0.000000
6	6	0	4.822345	0.243305	0.000001
7	1	0	4.863443	−1.925125	0.000005
8	1	0	2.417550	−2.152051	0.000005
9	1	0	1.990986	2.126185	0.000000
10	1	0	4.466158	2.360717	−0.000001
11	6	0	0.607909	−0.173821	0.000004
12	6	0	−0.599677	−0.288639	0.000006
13	6	0	−2.020383	−0.430250	−0.000002
14	6	0	−2.647289	−1.687303	0.000000
15	6	0	−2.834817	0.706151	−0.000002
16	6	0	−4.032606	−1.804022	−0.000001
17	1	0	−2.028865	−2.577823	0.000004
18	6	0	−4.216822	0.604303	−0.000003
19	6	0	−4.805185	−0.653396	−0.000004
20	1	0	−4.521905	−2.771495	0.000000
21	8	0	6.156345	0.479068	0.000001
22	9	0	−2.293228	1.928003	0.000000
23	9	0	−4.971381	1.706292	−0.000004
24	9	0	−6.142781	−0.722801	−0.000005
25	6	0	7.030569	−0.639938	0.000004
26	1	0	6.881556	−1.251688	−0.896030
27	1	0	8.039408	−0.230029	0.000004
28	1	0	6.881555	−1.251685	0.896039

Dipole moment (field-independent basis, Debye):

X = 4.2798 Y = −4.6934 Z = 0.0000 Tot = 6.3517

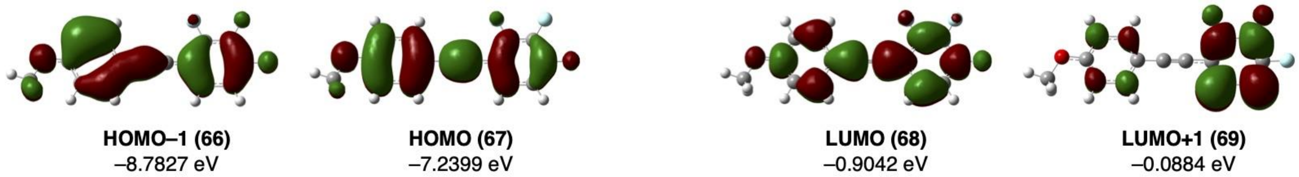

Excited State 1: Singlet-A 4.3115 eV 287.57 nm f = 1.3056 < S**2 > = 0.000

67 -> 68 0.68840

Geometry Optimization of **3Fb**

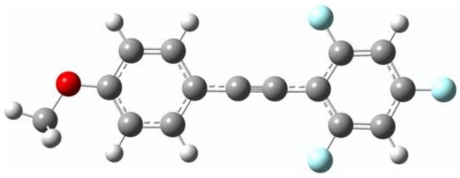

Center Number	Atomic Number	Atomic Type	Coordinates (Angstroms) X	Y	Z
1	6	0	3.912810	1.420533	−0.000033
2	6	0	2.530283	1.372193	−0.000031
3	6	0	1.858868	0.134633	−0.000003
4	6	0	2.615114	−1.043584	0.000024
5	6	0	4.007514	−1.003331	0.000021
6	6	0	4.660875	0.233568	−0.000007
7	1	0	4.440016	2.369330	−0.000056
8	1	0	1.955084	2.292951	−0.000052
9	1	0	2.108518	−2.003899	0.000046
10	1	0	4.563551	−1.933313	0.000042
11	6	0	0.428967	0.083412	−0.000001
12	6	0	−0.783062	0.042356	0.000000
13	6	0	−2.206255	−0.003912	0.000001
14	6	0	-2.989787	1.156941	0.000022
15	6	0	−2.914031	−1.212396	−0.000017
16	6	0	−4.373086	1.148560	0.000023
17	6	0	−4.295019	−1.292233	−0.000017
18	6	0	−4.991945	−0.092927	0.000003
19	1	0	−4.942662	2.070049	0.000040
20	1	0	−4.804649	−2.248190	−0.000032
21	9	0	−2.356925	2.337691	0.000041
22	9	0	−2.206997	−2.350333	−0.000037
23	9	0	−6.333925	−0.136208	0.000004
24	8	0	6.007168	0.384462	−0.000012
25	6	0	6.808688	−0.787640	0.000012
26	1	0	6.621171	−1.388757	−0.895995
27	1	0	7.841485	−0.442532	0.000002
28	1	0	6.621175	−1.388718	0.896045

Dipole moment (field-independent basis, Debye):

X = 1.9886 Y = −1.6513 Z = 0.0000 Tot = 2.5848

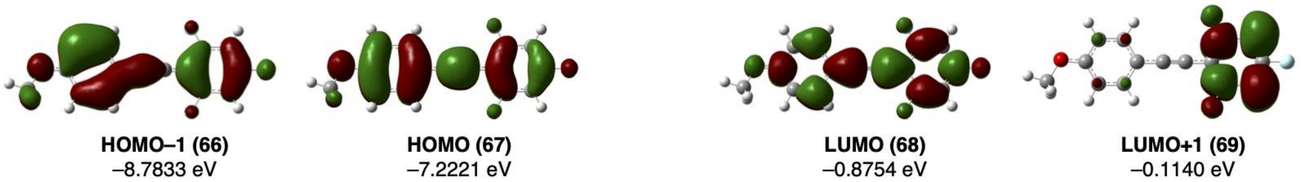

Excited State 1: Singlet-A 4.3203 eV 286.98 nm f = 1.3148 < S**2 > = 0.000

67 -> 68 0.68809

Geometry Optimization of **3Fc**

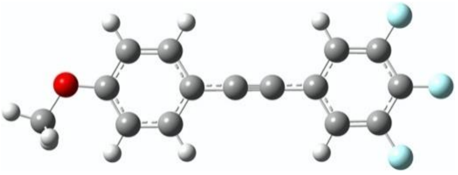

Center Number	Atomic Number	Atomic Type	Coordinates (Angstroms) X	Y	Z
1	6	0	4.239351	−1.418372	−0.000007
2	6	0	2.856701	−1.373859	−0.000004
3	6	0	2.180836	−0.138569	0.000003
4	6	0	2.934340	1.041571	0.000010
5	6	0	4.326851	1.005732	0.000008
6	6	0	4.983972	−0.229210	−0.000001
7	1	0	4.769398	−2.365592	−0.000013
8	1	0	2.284502	−2.296507	−0.000008
9	1	0	2.425150	2.000529	0.000017
10	1	0	4.880153	1.937354	0.000013
11	6	0	0.750486	−0.091554	0.000006
12	6	0	−0.462210	−0.054227	0.000006
13	6	0	−1.892552	−0.011794	0.000001
14	6	0	−2.628552	−1.207356	0.000001
15	6	0	−2.557727	1.224569	0.000001
16	6	0	−4.009573	−1.145456	−0.000002
17	1	0	−2.136608	−2.173364	0.000003
18	6	0	−3.940059	1.242958	−0.000001
19	1	0	−2.010445	2.160310	0.000003
20	6	0	−4.682714	0.069378	−0.000004
21	8	0	6.330567	−0.376083	−0.000004
22	6	0	7.128747	0.798371	0.000000
23	1	0	6.939478	1.398868	0.896041
24	1	0	8.162515	0.456220	−0.000004
25	1	0	6.939474	1.398878	−0.896033
26	9	0	−4.740938	−2.267894	−0.000003
27	9	0	−6.017055	0.108366	−0.000006
28	9	0	−4.604844	2.406071	−0.000001

Dipole moment (field-independent basis, Debye):

X = 5.8519 Y = 1.5465 Z = 0.0000 Tot = 6.0528

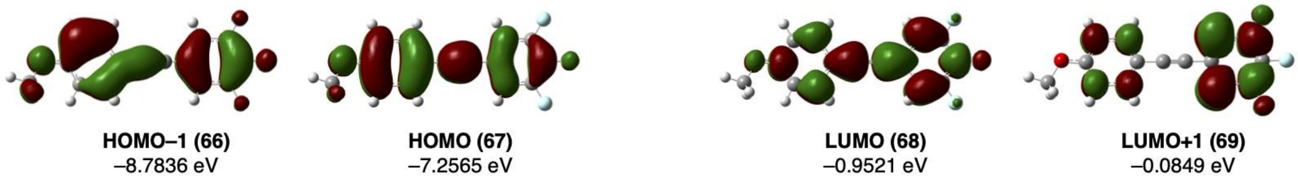

Excited State 1: Singlet-A 4.2847 eV 289.37 nm f = 1.2990 < S**2 > = 0.000

67 -> 68 0.68899

Geometry Optimization of **4F**

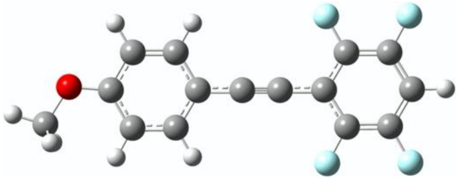

Center Number	Atomic Number	Atomic Type	Coordinates (Angstroms) X	Y	Z
1	6	0	−4.109753	−1.428299	−0.000009
2	6	0	−2.727870	−1.376550	−0.000007
3	6	0	−2.060942	−0.136358	0.000003
4	6	0	−2.819158	1.040833	0.000009
5	6	0	−4.210881	0.996742	0.000007
6	6	0	−4.860620	−0.242580	−0.000002
7	1	0	−4.634955	−2.378118	−0.000017
8	1	0	−2.149695	−2.295380	−0.000012
9	1	0	−2.314535	2.002130	0.000017
10	1	0	−4.769787	1.924921	0.000013
11	6	0	−0.632519	−0.081749	0.000005
12	6	0	0.579319	−0.039089	0.000006
13	6	0	2.001290	0.006309	0.000003
14	6	0	2.767765	−1.164265	0.000001
15	6	0	2.694446	1.221670	0.000001
16	6	0	4.152682	−1.115244	−0.000002
17	6	0	4.079845	1.257553	−0.000002
18	6	0	4.831155	0.093104	-0.000004
19	8	0	-6.205198	-0.396948	-0.000006
20	6	0	-7.011500	0.772437	0.000001
21	1	0	-6.826136	1.373816	0.896149
22	1	0	-6.826133	1.373829	-0.896139
23	1	0	-8.042738	0.422911	-0.000003
24	1	0	5.914585	0.126230	-0.000006
25	9	0	2.157998	-2.352753	0.000003
26	9	0	4.834220	-2.267936	-0.000004
27	9	0	2.012946	2.370568	0.000003
28	9	0	4.689439	2.449917	-0.000004

Dipole moment (field-independent basis, Debye):

X = -2.8011 Y = 1.6245 Z = 0.0000 Tot = 3.2381

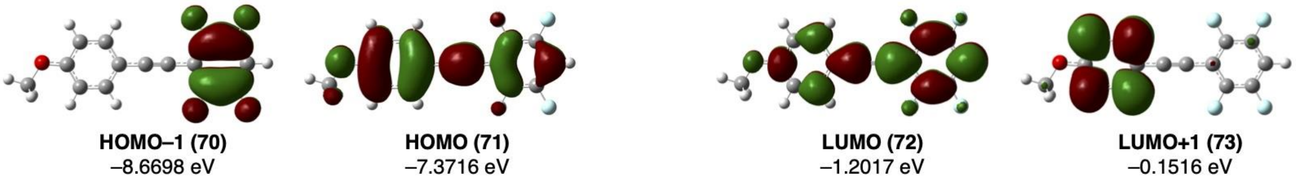

Excited State 1: Singlet-A 4.1966 eV 295.44 nm f = 1.2948 < S**2 > = 0.000

71 -> 72 0.68641

Geometry Optimization of **5F**

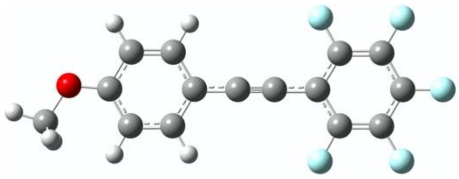

Center Number	Atomic Number	Atomic Type	Coordinates (Angstroms) X	Y	Z
1	6	0	4.446347	−1.423522	−0.000018
2	6	0	3.064247	−1.377319	−0.000016
3	6	0	2.392616	−0.139739	0.000001
4	6	0	3.146024	1.040479	0.000015
5	6	0	4.537909	1.001828	0.000014
6	6	0	5.192560	−0.234884	−0.000003
7	1	0	4.975258	−2.371276	−0.000030
8	1	0	2.489833	−2.298498	−0.000027
9	1	0	2.637700	1.999819	0.000028
10	1	0	5.093092	1.932239	0.000025
11	6	0	0.963938	−0.090322	0.000002
12	6	0	−0.247870	−0.051134	0.000002
13	6	0	−1.669520	−0.007913	0.000001
14	6	0	−2.435177	−1.179110	0.000005
15	6	0	−2.364445	1.206527	−0.000003
16	6	0	−3.820157	−1.146837	0.000004
17	6	0	-3.748955	1.256125	-0.000004
18	6	0	-4.479912	0.075262	-0.000001
19	8	0	6.537713	-0.383910	-0.000006
20	6	0	7.339319	0.788722	0.000008
21	1	0	7.151522	1.389352	-0.896135
22	1	0	7.151524	1.389330	0.896166
23	1	0	8.371952	0.443359	0.000002
24	9	0	-1.828150	-2.365735	0.000009
25	9	0	-4.522245	-2.279198	0.000007
26	9	0	-1.688310	2.355204	-0.000006
27	9	0	-4.382961	2.427991	-0.000009
28	9	0	-5.808792	0.114846	-0.000002

Dipole moment (field-independent basis, Debye):

X = 4.6676 Y = 1.5824 Z = 0.0000 Tot = 4.9286

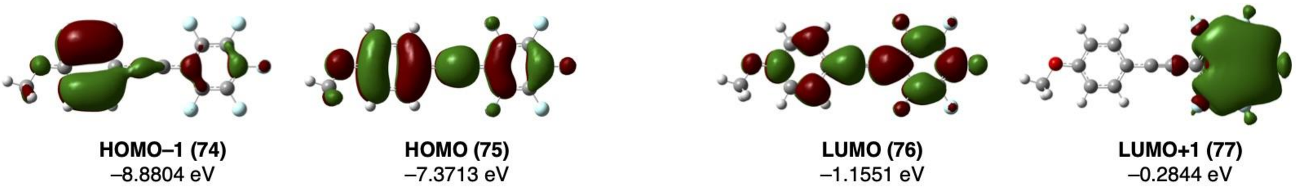

Excited State 1: Singlet-A 4.2313 eV 293.02 nm f = 1.3006 < S**2 > = 0.000

75 -> 76 0.68739
